# Supplementary material for: Causal modelling of variation in clinical practice and long-term outcomes of ADHD using Norwegian registry data: the ADHD controversy project
Source: BMJ Open. 2021 Jan 19;11(1):e041698. doi: 10.1136/bmjopen-2020-041698 (PMC7817799; doi:10.1136/bmjopen-2020-041698)
Supplement: Author's manuscript [file bmjopen-2020-041698.draft_revisions.pdf]

BMJ Open is committed to open peer review. As part of this commitment we make the peer review history of every article we publish publicly available.

When an article is published we post the peer reviewers' comments and the authors' responses online. We also post the versions of the paper that were used during peer review. These are the versions that the peer review comments apply to.

The versions of the paper that follow are the versions that were submitted during the peer review process. They are not the versions of record or the final published versions. They should not be cited or distributed as the published version of this manuscript.

BMJ Open is an open access journal and the full, final, typeset and author-corrected version of record of the manuscript is available on our site with no access controls, subscription charges or pay-per-view fees (http://bmjopen.bmj.com).

If you have any questions on BMJ Open's open peer review process please email info.bmjopen@bmj.com

BMJ Open

The ADHD controversy project
Study Protocol

Journal:	*BMJ Open*
Manuscript ID	bmjopen-2020-041698
Article Type:	Protocol
Date Submitted by the Author:	17-Jun-2020
Complete List of Authors:	Mykletun, Arnstein; University of Tromso Faculty of Health Sciences, Department of Community Medicine; Haukeland University Hospital, Center for Research and Education in Forensic Psychiatry<br>Widding-Havneraas, Tarjei; Haukeland University Hospital, Center for Research and Education in Forensic Psychiatry; University of Bergen, Department of Clinical Medicine<br>Chaulagain, Ashmita; Haukeland University Hospital, Center for Research and Education in Forensic Psychiatry; University of Bergen, Department of Clinical Medicine<br>Lyhmann, Ingvild; Haukeland University Hospital, Center for Research and Education in Forensic Psychiatry; University of Bergen, Department of Clinical Medicine<br>Bjelland, Ingvar; University of Bergen, Department of Clinical Medicine; Haukeland University Hospital, Department of Psychiatry<br>Halmøy, Anne; University of Bergen, Department of Clinical Medicine; Haukeland University Hospital, Department of Psychiatry<br>Elwert, Felix; University of Wisconsin-Madison, Department of Sociology<br>Butterworth, Peter; University of Melbourne<br>Markussen, Simen; Ragnar Frisch Centre for Economic Research,<br>Zachrisson, Henrik; University of Oslo, Department of Special Education<br>Rypdal, Knut; Haukeland University Hospital, Center for Research and Education in Forensic Psychiatry
Keywords:	Child & adolescent psychiatry < PSYCHIATRY, MENTAL HEALTH, EPIDEMIOLOGY

SCHOLARONE™
Manuscripts

I, the Submitting Author has the right to grant and does grant on behalf of all authors of the Work (as defined in the below author licence), an exclusive licence and/or a non-exclusive licence for contributions from authors who are: i) UK Crown employees; ii) where BMJ has agreed a CC-BY licence shall apply, and/or iii) in accordance with the terms applicable for US Federal Government officers or employees acting as part of their official duties; on a worldwide, perpetual, irrevocable, royalty-free basis to BMJ Publishing Group Ltd ("BMJ") its licensees and where the relevant Journal is co-owned by BMJ to the co-owners of the Journal, to publish the Work in this journal and any other BMJ products and to exploit all rights, as set out in our licence.

The Submitting Author accepts and understands that any supply made under these terms is made by BMJ to the Submitting Author unless you are acting as an employee on behalf of your employer or a postgraduate student of an affiliated institution which is paying any applicable article publishing charge ("APC") for Open Access articles. Where the Submitting Author wishes to make the Work available on an Open Access basis (and intends to pay the relevant APC), the terms of reuse of such Open Access shall be governed by a Creative Commons licence – details of these licences and which Creative Commons licence will apply to this Work are set out in our licence referred to above.

Other than as permitted in any relevant BMJ Author's Self Archiving Policies, I confirm this Work has not been accepted for publication elsewhere, is not being considered for publication elsewhere and does not duplicate material already published. I confirm all authors consent to publication of this Work and authorise the granting of this licence.

For peer review only

The ADHD controversy project

Study Protocol

Arnstein Mykletun[1,2,3,4], Tarjei Widding-Havneraas[2,5], Ashmita Chaulagain[2,5], Ingvild Lyhmann[2,5], Ingvar Bjelland[5,6], Anne Halmøy[5,6], Felix Elwert[7], Peter Butterworth[8,9], Simen Markussen[10], Henrik Daae Zachrisson[11], Knut Rypdal[2]

[1] UiT - The Arctic University of Norway, Tromsø, Norway

[2] Center for Research and Education in Forensic Psychiatry, Haukeland University Hospital, Bergen, Norway

[3] Division of Mental Health, Norwegian Institute of Public Health, Oslo, Norway

[4] Nordland Hospital Trust, Centre for Work and Mental Health, Bodø, Norway

[5] Department of Clinical Medicine, University of Bergen, Norway

[6] Department of Psychiatry, Haukeland University Hospital, Bergen, Norway

[7] Department of Sociology, University of Wisconsin-Madison, WI, USA

[8] Research School of Population Health, Australian National University, Canberra, Australia

[9] Melbourne Institute of Applied Economic and Social Research, University of Melbourne, Melbourne, Australia

[10] Ragnar Frisch Centre for Economic Research, Oslo, Norway

[11] Department for Special Education, University of Oslo, Oslo, Norway

For peer review only - http://bmjopen.bmj.com/site/about/guidelines.xhtml

Corresponding author: Arnstein Mykletun: arnstein.mykletun@uit.no

Tarjei Widding-Havneraas: tarjei.widding-havneraas@helse-bergen.no

Ashmita Chaulagain: ashmita.chaulagain@helse-bergen.no

Ingvild Lyhmann: ingvild.lyhmann@helse-bergen.no

Ingvar Bjelland: ingvar.bjelland@uib.no

Anne Halmøy: anne.halmoy@uib.no

Felix Elwert: elwert@wisc.edu

Peter Butterworth: peter.butterworth@unimelb.edu.au

Simen Markussen: simen.markussen@frisch.uio.no

Henrik Daae Zachrisson: h.d.zachrisson@isp.uio.no

Knut Rypdal: knut.rypdal@helse-bergen.no

Word count: 5236 (excluding abstract and reference).

For peer review only

Abstract

Introduction: Attention-Deficit/Hyperactivity Disorder (ADHD) is among the most common mental disorders in children and adolescents, and it is a strong risk factor for several adverse psychosocial outcomes over the lifespan. There are large between- and within-country variations in diagnosis and medication rates. Due to ethical and practical considerations, few studies have examined the effects of receiving a diagnosis, and there is a lack of research on effects of medication on long-term outcomes.

Our project has four aims organized in four work packages: (WP1) To examine the prognosis of ADHD (with and without medication) compared to patients with other psychiatric diagnoses, patients in contact with public sector child and adolescent psychiatric outpatient clinics (without diagnosis), and the general population; (WP2) Examine within-country variation in ADHD diagnoses and medication rates by clinics' catchment area; (WP3) Identify causal effects of being diagnosed with ADHD and (WP4) ADHD medication on long-term outcomes.

Method and analysis: Our project links several nationwide Norwegian registries. The patient sample is all persons aged 5-18 years that were in contact with public sector child and adolescent psychiatric outpatient clinics in 2009-2011. Our comparative analysis of prognosis will be based on survival analysis and mixed-effects models. Our analysis of variation will apply mixed-effects models and generalized linear models. We have two identification strategies to retrieve the effect of being diagnosed with ADHD and of receiving medication on long-term outcomes. Both strategies rely on using preference-based instrumental variables, which in our project are based on provider preferences for ADHD diagnosis and medication.

Ethics and dissemination: The project is approved by the Regional Ethics Committee, Norway (REC number 2017/2150/REC south-east D). All papers will be published in open access journals and results will be presented in national and international conferences.

For peer review only - http://bmjopen.bmj.com/site/about/guidelines.xhtml

Trial registration: Submitted and waiting for reply**.**

This is an open access article distributed in accordance with the Creative Commons Attribution Non Commercial (CC BY-NC 4.0) license, which permits others to distribute, remix, adapt, build upon this work non-commercially, and license their derivative works on different terms, provided the original work is properly cited, appropriate credit is given, any changes made indicated, and the use is non-commercial. See: http://creativecommons.org/licenses/by-nc/4.0/.

Strengths and limitations of this study:

- This study will use national registry-based data to study real-world, long-term effects of ADHD diagnosis and medication on psychosocial outcomes.
- The identification strategies will provide answers that RCTs cannot provide for ethical and practical reasons.
- To our knowledge, our project will be the first application of diagnostic variation as an instrument variable used to estimate the causal effect of diagnosis on long-term outcomes.
- The main limitation of registry-based research is that data are limited to information collected by registries.

For peer review only - http://bmjopen.bmj.com/site/about/guidelines.xhtml

For peer review only

Introduction

Attention-Deficit/Hyperactivity Disorder (ADHD) is among the most common mental disorders in children and adolescents (1-4), which is often associated with problems into adulthood (5). ADHD is a persistent neurodevelopmental disorder characterized by symptoms of inattention and/or hyperactivity and impulsivity that are excessive for an individual's age or overall development (6, 7). ADHD is a strong risk factor for school dropout (8), injuries (9), comorbidity (10), self-harm and mortality (11-14), contact with child welfare services (15, 16), as well as lack of employment (17, 18), later substance use disorders (17, 19-21) and criminality (22-24).

Clinical treatment guidelines recommend a multimodal approach that combine psychosocial and pharmacological treatment options (25, 26). *The Multimodal Treatment of Attention Deficit Hyperactivity Disorder Study* (MTA) (27-30) has been influential in shaping treatment guidelines. The main conclusions from MTA was that combined behavioral therapy and medication, and medication alone, was superior to behavioral therapy and community care alone. However, MTA was published in 1999, before the now standard extended-release capsules were introduced, and 14 months follow-up in one sample is not conclusive of long-term treatment effects (31). Short-term studies show that medication reduces symptoms in at least 75 % of children and adolescents with ADHD (32), suggesting that medication may be superior to both psychological interventions and no treatment (33). While short-term effects of medication are established (although with some discussions surrounding the evidence base) (34), there are few studies on long-term effects of medication. One recent randomized double-blinded placebo-controlled discontinuation trial showed that medication was generally effective in reducing symptoms for approximately 40 % of individuals after two years (35).

More long-term studies that examine functional outcomes in real-world settings are needed (36). A promising source of long-term effect estimates is nationwide register studies (5, 31). In

For peer review only - http://bmjopen.bmj.com/site/about/guidelines.xhtml

recent years, register studies using quasi-experimental designs have shown that medication may reduce risk of crime (37-40), substance abuse (41), injuries (9, 42), and motor vehicle accidents (43).

Over recent decades there have been substantial increases in clinical diagnosis and pharmacological treatment of ADHD in many countries (2), including North America (44), Europe (45), Australia (46), and Asia (47). Recent systematic reviews and meta-analyses estimate that the global prevalence rate of ADHD in children and adolescents is between 3.4 % (48) and 7.2 % (49). It has been estimated that at least an additional 5 % of children and adolescents have substantial difficulties with excessive and impairing levels of over-activity, inattention and impulsivity that are just below the threshold for diagnostic criteria (50). Some studies attribute the variation in prevalence estimates to social and cultural factors (51), while others downplay geographical location and argue for a methodological explanation (3).

A retrospective study based on population-level databases from 13 countries and 154.5 million individuals showed that prevalence of ADHD medication use in children aged 3-18 years varied between 0.27 % and 6.69 % across included countries (52). Among the Nordic countries, the prescription prevalence has been estimated to range from 0.12 % in Finland to 1.25 % in Iceland (53). Further, large within-country variation in prescription rates has been reported in several countries, e.g. the USA (54-56), Denmark (57, 58), Sweden (59), and Germany (60).

Even within Norway, a country with a modest inequality and a universal healthcare system, there are substantial regional differences in diagnosis and medication (61, 62). For instance, the county-wise rates for ADHD diagnosis in Norway at ages 6-12 vary between 1.7 % to 4.8 % for boys and between 0.4 % to 2.0 % for girls, and similar variation has been noted for medication rates (61). In total, 80 % of the children and adolescents diagnosed with ADHD fill one prescription for ADHD medication (61), and approximately 50 % continue to collect

For peer review only - http://bmjopen.bmj.com/site/about/guidelines.xhtml

prescriptions for more than one year (63). In addition to geographical variation in diagnosis and medication rates, studies have shown that being young relative to peers is a strong predictor for being diagnosed with ADHD in several countries (64, 65), despite ADHD being an underlying neurodevelopmental disorder. A Norwegian chart review indicated that only 49 % of all diagnoses were adequately documented (66).

Internationally, the variation in ADHD diagnosis and medication rates have caused a debate with two opposing perspectives. On the one hand, there is a restrictive perspective characterized by concerns about potential overdiagnosis of ADHD, leading to medicalization of normal behavior (67), unnecessary stigma (68), and side-effects of pharmacological treatment (69) that might not even be helpful in the presence of true ADHD. On the other hand, there is a liberal perspective distinguished by concerns about underdiagnosis and undertreatment (70), which argues that increased rates of ADHD diagnosis and medication are due to improved recognition and understanding by professionals (71, 72), broadened diagnostic definitions (73), and methodological problems in the literature (2). This perspective emphasizes the importance of reducing the risk of adverse outcomes associated with untreated ADHD, such as injuries (9), school dropout (8), and criminal behavior (42).

This debate may be reflected in clinical practice, where patients referred to specialist healthcare with suspicion of ADHD present with various degrees and compositions of symptoms (Figure 1). Clinicians generally regard ADHD symptoms on a continuum. Although the diagnostic manuals define a symptomatic and functional cut-off for ADHD (74), diagnosis is inevitably based on a clinical judgement. This discretionary decision is likely to vary between clinicians (61), partly due their adherence to a restrictive or liberal perspective. Figure 1 presents our theoretical model on clinician's consensus on diagnosis and medication by ADHD symptom load among patient's in contact with child and adolescent psychiatric outpatient clinics. Clinicians will generally agree that patients with very few or no symptoms of ADHD should

For peer review only - http://bmjopen.bmj.com/site/about/guidelines.xhtml

receive neither a diagnosis nor medication (left side of Figure 1) (75-77). On the opposite end of the continuum are patients with obvious ADHD symptoms, for whom most if not all clinicians will agree on ADHD diagnosis and medication (right side of Figure 1).

Figure 1. Theoretical model on clinician's consensus on diagnosis and medication by ADHD symptom load among referred patients.

The controversy on ADHD diagnosis and ADHD medication mostly relates to the middle area of the symptom distribution in Figure 1, which includes patients with ambiguous symptoms, bordering on the threshold for diagnostic cut-off. These patients may be presenting with symptoms compatible also with other diagnoses or could be experiencing psychosocial stress that may produce ADHD-like symptoms, making an ADHD diagnosis difficult to establish.

In light of increasing ADHD diagnosis and medication rates in most Western countries, it is important to improve our knowledge on the effect of diagnosis among patients around the clinical threshold for diagnosis, as well as the effects of medication on long-term outcomes among patients. This is an important issue to address as it can inform the debate on under- and overdiagnosis and medication. A pressing issue is whether patients with ambiguous ADHD symptoms benefit or not from being diagnosed. As pointed out by Owens (78), an ADHD diagnosis "… can bring beneficial pharmacological treatment and social supports, but it can also trigger negative social and psychological processes, as suggested by labeling theory". Being diagnosed entails a set of at least three components that interact on long-term outcomes: (i) stigma ("diagnosis labeling effects") (75, 79-82), (ii) medication, and (iii) psychosocial treatment. Owens (78) finds that consistent with labeling theory (stigma), only those with severe

For peer review only

For peer review only - http://bmjopen.bmj.com/site/about/guidelines.xhtml

pre-diagnosis symptoms seem to benefit from diagnosis and medication, while those with mild pre-diagnosis symptoms have worse outcomes compared to undiagnosed matches.

The key issue in identifying an effect of receiving a diagnosis is that diagnoses cannot be randomized to patients. Hence, we must either rely on epidemiological designs that precludes causal inference unless all potential confounding is statistically controlled, or we need to find sources of plausibly exogenous variation that can be used as a natural experiment. We believe the geographical variation in diagnosis and medication can be used as a natural experiment to identify causal effects of diagnosis and medication on long-term outcomes. There are several plausible explanations for geographical variation in diagnosis and medication rates on both the supply and demand side, including resources, capacity, and patient mix. Nevertheless, a portion of the variation may be due to variation in clinician's diagnosis and medication practice among public sector child and adolescent psychiatric outpatient clinics, commonly referred to as provider preference (83), which can lead to variation in diagnosis and prescription decisions for assumed similar cases. This portion can provide natural experiments in treatment decisions using provider preference as an instrumental variable (84). Considering the regional differences in ADHD diagnosis and treatment, we expect that the child and adolescent outpatient clinics in Norway have different preferences (Figure 3). In single-provider universal access health systems, variation in health service provision that is not accounted for by differences in patient populations and symptom load is usually regarded as unwarranted and unwanted (85).

The current research project aims to contribute to the existing knowledge on ADHD diagnosis and ADHD medication in several ways. Our project will employ Norwegian registry data to explore four main aims with corresponding work packages:

WP1: The epidemiology of ADHD prognosis: We will examine the prognosis of patients diagnosed with ADHD in child and adolescent psychiatry (with and without medication). These

For peer review only - http://bmjopen.bmj.com/site/about/guidelines.xhtml

will be compared to patients in child and adolescent psychiatry who received other diagnoses, or who received no diagnosis. We will also compare to the general population without contact with child and adolescent psychiatry. Measures of prognosis include registry data records of emergency care visits/injuries, comorbidity, contact with child welfare services, contact with adult psychiatric health services, education, employment and income, welfare dependency, crime, self-harm, suicide, and mortality.

WP2: The ADHD variation controversy: We will describe the variation in clinical practice between about 100 catchment areas for child and adolescent psychiatric outpatient clinics in Norway in both the rate of ADHD diagnosis (as a proportion of all children referred to these clinics) and the rate of ADHD medication (as a proportion of those diagnosed with ADHD). We will explore to what extent this variation in clinical practice can be attributed to catchment area characteristics.

WP3: Long-term effects of receiving an ADHD diagnosis. We will identify causal effects of being diagnosed with ADHD for among patients in contact with child and adolescent psychiatric outpatient clinics on long-term outcomes including emergency care visits/injuries, comorbidity, education, contact with child welfare services, contact with adult psychiatric health services, employment and income, welfare dependency, crime, self-harm, suicide, and mortality.

WP4: Long-term effects of ADHD medication. We will identify causal effects of initial ADHD medication among patients diagnosed with ADHD on long-term outcomes including emergency care visits/injuries, comorbidity, education, contact with child welfare services, contact with adult psychiatric health services, employment and income, welfare dependency, crime, self-harm, suicide, and mortality.

For peer review only - http://bmjopen.bmj.com/site/about/guidelines.xhtml

Methods and analysis

Design

Our project employs methods for descriptive analyses and causal inference (86, 87). Our study draws on several linked Norwegian nationwide registries. Nordic registry data provide individual level data for the whole population (88), and are only censored by migration and death (89). Our project has four work packages:

WP1: The epidemiology of ADHD prognosis

We will use survival analysis (90) to examine time-to-event for our outcomes. In line with recent studies (91), we will account for possible competing risks to avoid overestimation of the cumulative incidence of our events of interest. We will also apply mixed-effects analysis, which is well-suited and an established standard for individual-level longitudinal data with repeated observations (90, 92).

WP2: The ADHD variation controversy

To account for patient clustering and repeated observations over time, we will use standard generalized linear models with cluster-robust standard errors and mixed-effects models (90, 93).

WP3 and WP4: Long-term effects of receiving an ADHD diagnosis and ADHD medication

To identify the effect of ADHD diagnosis (WP3) and ADHD medication (WP4) on long-term outcomes, we will use child and adolescent psychiatric outpatient clinics' catchment area variation in diagnosis and medication as instrumental variables (IV). IV is a common identification strategy in health-services research (99), that exploits the variation induced in a treatment variable (here, ADHD diagnosis or ADHD medication prescription) by a plausibly exogenous (as-if random) event or process (i.e., the instrument). The role of the instrument is to "isolate" exogenous variation in the treatment, and hence to remove the endogenous

For peer review only - http://bmjopen.bmj.com/site/about/guidelines.xhtml

For peer review only

(confounded) variation, so that the analysis only uses the exogenous variation to estimate the treatment effect (94). In this sense, IV analysis is a quasi-experimental approach. There is evidence to support that research designs with valid IVs can provide similar estimates as randomized experiments on the same research questions (95-97), whereas more traditional observational studies without a credible source of exogenous variation often do not (94, 97). Our identification strategies exploit as-if random variation in providers' preference to diagnose patients with ADHD and to prescribe ADHD medication (42, 98) across Norwegian public sector child and adolescent psychiatric outpatient clinics. The main idea behind provider preference-based instruments is that "…different providers or groups of providers have different preferences dictating how medications or medical procedures are used." (83). If the provider preference is associated with treatment, does not directly affect the outcome, and is not associated with unmeasured risk factors of the outcome, it can be used as an IV to consistently estimate the treatment effect (84). We will use two candidate provider preference instruments, illustrated in Figure 2.

Figure 2. Two sources of as-if randomization. Quasi-experiment 1 is provider preference for ADHD diagnosis. Quasi-experiment 2 is provider preference for ADHD medication among those diagnosed with ADHD.

Instruments

It is plausible that a provider's preference for diagnosing ADHD affects whether or not an individual is diagnosed with ADHD, while it is not associated with the outcomes of interest (i.e. via paths other than affecting diagnosis) net of certain controls. We will use provider preference for ADHD diagnosis to identify the effect of diagnosis on long-term outcomes.

For peer review only - http://bmjopen.bmj.com/site/about/guidelines.xhtml

Similarly, provider preference for ADHD medication likely affects whether or not a patient receives medication, while it is not associated with our outcomes of interest, net of certain controls. Provider preference for ADHD medication will be used to identify the effect of ADHD medication on long-term outcomes. Provider preferences for medication have become a well-established IV in epidemiology (99), as they are usually strongly associated with medication use and reduce covariate imbalance (100). Our IVs will be defined at the provider-level (i.e. the catchment area of child and adolescent psychiatric outpatient clinics), which has been shown to be stronger and more efficient compared to physician-level instruments (101). The (catchment) area definition of child and adolescent psychiatric outpatient clinics strikes a reasonable balance between methodological issues introduced by too large or too small area definitions (102).

To our knowledge, provider-level preference for diagnosis (61, 62) is a novel candidate instrument, while provider preference for ADHD medication has only been used in at least four prior studies (42, 98, 103, 104), which do not cover all outcomes in our project. We illustrate the variation that will be used as provider preference instruments for the effects of diagnosis (WP3) and medication (WP4) at the county level in Figure 3. Our eventual analysis will use preferences measured at the level of catchment areas of child and adolescent psychiatric outpatient clinics.

Figure 3. County variation in ADHD diagnosis and medication percent for children and adolescents between 10 to 19 years in Norway. ADHD as main diagnosis once or more times during 2009-2011.

For peer review only

Although rates of diagnosis and medication follow each other closely, preliminary evidence on the county level indicates that they do not correlate perfectly, as shown in Figure 3 and Figure 4.

Figure 4. Scatterplot of ADHD diagnosis and medication among children and adolescents between 10 to 19 years by Norwegian counties. ADHD as main diagnosis once or more times during 2009-2011. Linear fitted line.

Setting of study and assessment of identification strategy

Preference-based instruments may lead to biased inferences if the identifying assumptions are not met (105). The application of IV rests on a set of key assumptions that must to warrant a credible causal interpretation of estimates. An IV-strategy can be evaluated on a continuum from low to high credibility based on the underlying assumptions (106). The two main assumptions of IV are relevance and exclusion. Relevance can be tested statistically, while exclusion can only be partially tested and relies on justification derived from subject-matter knowledge. The instruments are likely to meet the relevance assumptions as public sector child and adolescent psychiatric outpatient clinics are the only institutions in Norway that can diagnose ADHD and initiate pharmacological treatment of ADHD (aside from a small number of private practices), and the share of patients diagnosed with ADHD and medicated for ADHD varies considerably across areas. We expect that the instrument meets the exclusion restriction in several respects. We expect provider preference for diagnosis and medication only to affect long-term outcomes through diagnosis and treatment decisions. Although patients in Norway are free to choose outpatient clinics, which opens the door to self-selection bias in principle, in practice, the vast majority of patients uses the nearest child and adolescent psychiatric

For peer review only - http://bmjopen.bmj.com/site/about/guidelines.xhtml

outpatient clinics for convenience and because child and adolescent psychiatric outpatient clinics are expected to deliver equal services of equal quality (107). There is no competitive market in this part of the health sector. Furthermore, it is unlikely that patients would sort in response to provider preferences, as the variation in rates of ADHD diagnosis and treatment are not common knowledge among patients or their patients. Consequently, children with similar ADHD symptoms are *as-if* randomly assigned to ADHD medication or no ADHD medication at a national level. Next, we will control for the characteristics of the catchment area and the patient mix of each provider that may partially explain variation in diagnosis and prescription practices. Finally, exclusion is plausible due to the existence of large geographical variation in ADHD diagnosis and medication, net of demographic factors (42, 61, 108), which suggests that clinical practice varies for idiosyncratic reasons.

Triangulation and statistical methodology

Our assessment of causal effects relies on IV triangulated with other methods based on other assumptions (109). As pointed out by Matthay et al. (110): "All methods involve untestable assumptions and tradeoffs in precision and validity" and there is seldom a "… clear justification for exclusive reliance on one method". We will supplement our IV-analysis with mixed-effects models, propensity score matching (PSM), inverse-probability weighting and fixed-effects regression (for medication) (93). These are also the methods we will base our analysis on if IV-conditions are not met. All analyses will be performed in Stata (111) and R (112).

Data and study sample

Our data comprises one sample of patients and one sample of controls from the general population. The patient sample includes all persons between 5 and 18 years of age that had any contact (for any reason) with child and adolescent psychiatric outpatient clinics between 2009-2011 (i.e. birth cohorts 1991-2006). Children younger than 5 years are not included as few

For peer review only - http://bmjopen.bmj.com/site/about/guidelines.xhtml

children are diagnosed with ADHD before age 5 (42). The control sample is a random sample from the general population of equal size as the patient sample defined as individuals in Norway 5-18 years old without reported contact (for any reason) with child and adolescent psychiatric outpatient clinics in 2009-2011. The control sample is included to enable us to compare the prognosis among patients diagnosed with ADHD and patients in contact with child and adolescent psychiatric outpatient clinics to the general population.

As the project will examine outcomes that occur at different points over a life course (injuries, contact with child welfare services, contact with adult psychiatric health services, education, income, welfare dependency, comorbidity, crime, self-harm, suicide, and mortality), we will receive data for 2009-2019 in 2020 and for 2020-2021 in 2022. We have estimated that the patient sample with an equally sized control sample from the general population (matched on age, gender and geography) will be approximately 200 000. All analyses are registry-based. We combine data from a total of nine national registries, presented in Table 1. (Abbreviations in parentheses.)

Table 1. Data from registry data included in the project

National register	Variables
Norwegian Patient Register (NPR)	Diagnoses (ICD-10, all F-diagnoses by multiaxial coding), institution, in/out date, waiting time code, contact type, referral from institution, referral type, referral reason, injury (specialist care), injury severity, child welfare service involvement, child's care situation, child's environment, gender, age, patient catchment area
Control and Payment of Health Reimbursement Register (KUHR)	Injuries (emergency room) ICPC-2 diagnoses (somatic: fracture, concussion, eye injuries, penetration, burn, poisoning, other injuries/ psychiatric: suicide attempt, hyperkinetic disorder, anorexia, schizophrenia)

For peer review only - http://bmjopen.bmj.com/site/about/guidelines.xhtml

Norwegian Cause of Death Registry (NCoDR)	Death, cause of death (three categories: suicide, injury, other)
Norwegian Prescription Database (NorPD)	Psychostimulants for ADHD (ATC-codes N06B, CO2, CO2A), melatonin (ATC-codes N05, N05B), antidepressants (ATC-code N06A), antiepileptics (ATC-code N03A), defined daily dose (DDD)
The Central Penal and Police Register (Strasak)	Criminal charges (ten categories: property theft, other offences for profit, criminal damage, violence and maltreatment, sexual offenses, drug and alcohol offences, public order and integrity violations, traffic offences, other offences)
The Central Population Register (CPR)	Country of origin (world region), parents' marital status, emigration
Municipality-State-Reporting (KOSTRA)	Case type, who reported case, content of case, measure in child welfare services law, removal of custody, reason for administrative decision
Norwegian Education Database (NUDB)	Highest completed level of education, parents' educational level, ended high school education before completion ("dropout"), average grade level for primary school, grades from high school completion, national test scores, order/conduct, absence
Income, tax and wealth register	Income, work assessment benefit (AAP), social assistance, disability pension benefit, unemployment assistance ("dagpenger"), parents' income

The registry data set will be supplemented with a municipality data set based on publicly available aggregated administrative data. The municipalities will be grouped by child and adolescent psychiatric outpatient clinic-catchment areas. The municipality data set serves two purposes: (i) examine associations between environmental factors and geographical variation in ADHD diagnosis and prescription patterns, and (ii) account for population mix at child and adolescent psychiatric outpatient clinics in analyses. The municipality data set will include covariates for socioeconomic conditions (education, income, welfare recipiency), crime,

For peer review only

income inequality, living conditions, health service utilization, and health service economic variables.

Patient and public involvement

Patients and public were not involved in the development of work packages or study design.

Discussion

This project examines (i) the prognosis of ADHD, (ii) within-country variation in ADHD diagnosis and medication rates by clinics' catchment area, (iii) causal effects of being diagnosed with ADHD on long term outcomes and (iv) causal effects of ADHD medication on long-term outcomes. The project relies on Norwegian nationwide register data and employs several methodological approaches, including survival analysis, mixed-effects models, and instrumental variables.

The instrumental variables analyses can provide estimates on effects of ADHD diagnosis, and effects of ADHD medication among patients that are diagnosed with ADHD and that receive may receive ADHD medication conditioned on provider preference for medication. In this sense, this project has merit to provide empirical evidence that neither RCTs nor conventional epidemiological designs can address. First, there is to our knowledge no trail randomizing children to ADHD diagnosis. Our approach aims to fill this knowledge gap by instrument variable analysis for children at the margin of ADHD diagnosis. Second, most trials of medication in ADHD to date has been conducted on children that clearly meet diagnostic criteria, whereas the variation and controversy is regarding children on the margin of receiving medication. RCTs on ADHD do not address this controversy. Our approach aims to fill this knowledge gap by instrument variable analysis for children diagnosed with ADHD on the margin of receiving treatment.

For peer review only - http://bmjopen.bmj.com/site/about/guidelines.xhtml

These studies would not have been feasible due to the long follow-up required. Randomizing would also be difficult from an ethical perspective, and recruitment to such a study would also be challenging.

Causal inference from conventional epidemiological designs is generally implausible due to unobserved confounding. By combining rich nationwide register data with an instrumental variables design, we are able to circumvent the shortcomings of both RCTs and conventional epidemiological designs on these topics.

This project is the first detailed examination of within-country variation in ADHD diagnosis and ADHD medication by child and adolescent outpatient psychiatric clinics' catchment areas, including possible associations with socioeconomic and demographic covariates. The project will be the first application of provider preference for ADHD diagnosis as an instrumental variable. The project will also be the first application of instrumental variables to assess the effects of ADHD diagnosis and ADHD medication on several psychosocial outcomes.

The project is subject to the following limitations. Register data is limited to pre-collected data in registers, which also determine data selection and data quality (113). Register data can be considered as the "administrator's view of the world" which affects data definitions and data collection practice, and often makes it challenging to understand data-generating processes (113). Moreover, register data are generally considered reliable, but the validity is important to assess due to complex data collection processes that varies between registers (89, 114).

Ethics and dissemination

Data management

For peer review only - http://bmjopen.bmj.com/site/about/guidelines.xhtml

The Norwegian Prescription Database (NorPD) will combine data from the different registries and replace the personal identification number with a project-specific number (pseudonymization) before distributing data to the research group to protect the privacy of participants. NorPD will keep the linkage key between the personal identification number and the project-specific number. Data will be stored and analyzed on a secure server at Haukeland University Hospital that only the PI and other pre-approved researchers will have access to. To ensure the quality and reproducibility of our data management and statistical analyses, we will (i) cross-check each step in the data cleaning and preparation process, and (ii) document each step from the raw data to the final data sets and statistical analyses in version-controlled syntax and log-files. Missing data will be assessed, and we will consider strategies for multiple imputation.

Ethics approval and consent to participate: The project is approved by the Regional Committee for Medical and Health Research Ethics, Norway: REC South East, Committee D (REC number 2017/2150/REC south-east D). Due to the large number of participants it is not feasible to obtain consent from all participants and is thus approved without this requirement. The study has been evaluated by REC to be "of considerable importance to society" and to "protect the integrity and welfare of the participants". Based on the fulfillment of these criteria, the project has been exempted from the requirement of collecting informed consent from participants. The REC approval is given under the precondition that the project is conducted as described in the project application, protocol, communication, and the administrative provisions in the Norwegian Health Research Act ("Helseforskningsloven") and appurtenant regulations.

Availability of data and materials: The data used in this project may not be made publicly available and is accessed due to the sensitive nature of the data. Current approvals and regulations do not allow sharing these data. Due to personal data protection regulations

For peer review only - http://bmjopen.bmj.com/site/about/guidelines.xhtml

(GDPR), restrictions in the ethics approval and restrictions by the registry owners, they may not be shared. We may share statistical code.

Dissemination: The publication strategy distinguishes between six types of publications planned from this project:

a. Preparatory publications: This includes the present protocol paper, trial registrations, systematic literature reviews on both subject matter issues regarding ADHD and provider-preference IV applications, and a survey on attitudes and clinical practice with regard to ADHD among clinicians currently working in child and adolescent psychiatric outpatient clinics.
b. WP1: The epidemiology of ADHD prognosis: Will utilize all data to describe common trajectories in ADHD. Multiple comparison groups will be employed including other patients referred to child and adolescent psychiatry who got other diagnoses or no diagnosis, and also the general population. Outcomes are according to data as described in Table 1.
c. WP2: The variation in ADHD diagnosis and treatment: We will describe in detail the geographical variation in ADHD diagnosis and medication, and also explore if characteristics with the catchment areas may explain some of the observed variation in clinical practice.
d. WP3: Long-term effects of receiving an ADHD diagnosis for children on the margin. Publications on the effect of ADHD diagnosis among those referred to child and adolescent psychiatry on outcomes as described above. Outcomes according to data in Table 1.
e. WP4: Long-term effects of ADHD medication. Publications on the effect of ADHD medication among those diagnosed with ADHD on outcomes as described above. Outcomes according to data in Table 1.

For peer review only - http://bmjopen.bmj.com/site/about/guidelines.xhtml

f. Exploratory publications on hypotheses and topics beyond those here described. These may be subject to additional approvals from the ethics committee and the owners of the registries, eventually also an update of the Data Protection Impact Assessment.

We will follow International Committee of Medical Journal Editors (ICMJE) Vancouver recommendations for authorship when publishing the papers. Most of our team members have significant experience in writing and publishing scientific papers, and we believe our multidisciplinary team's experience will serve as the main expertise in writing the planned articles. We aim to publish with open access. Substantial amendments to our protocol will be reported to relevant parties through relevant channels.

For peer review only

For peer review only - http://bmjopen.bmj.com/site/about/guidelines.xhtml

References

1. Polanczyk GV, Salum GA, Sugaya LS, Caye A, Rohde LA. Annual research review: A meta-analysis of the worldwide prevalence of mental disorders in children and adolescents. J Child Psychol Psychiatry. 2015;56(3):345-65.

2. Polanczyk GV, Willcutt EG, Salum GA, Kieling C, Rohde LA. ADHD prevalence estimates across three decades: an updated systematic review and meta-regression analysis. International Journal of Epidemiology. 2014;43(2):434-42.

3. Polanczyk G, de Lima MS, Horta BL, Biederman J, Rohde LA. The Worldwide Prevalence of ADHD: A Systematic Review and Metaregression Analysis. Am J Psychiat. 2007;164(6):942-8.

4. Thomas R, Sanders S, Doust J, Beller E, Glasziou P. Prevalence of attention-deficit/hyperactivity disorder: a systematic review and meta-analysis. Pediatrics. 2015;135(4):e994-1001.

5. Franke B, Michelini G, Asherson P, Banaschewski T, Bilbow A, Buitelaar JK, et al. Live fast, die young? A review on the developmental trajectories of ADHD across the lifespan. European neuropsychopharmacology : the journal of the European College of Neuropsychopharmacology. 2018;28(10):1059-88.

6. American Psychiatric Association. Diagnostic and statistical manual of mental disorders : DSM-5. 5th ed. ed. Washington, D.C: American Psychiatric Association; 2013.

7. Faraone SV, Asherson P, Banaschewski T, Biederman J, Buitelaar JK, Ramos-Quiroga JA, et al. Attention-deficit/hyperactivity disorder. Nat Rev Dis Primers. 2015;1:23.

8. Shaw M, Hodgkins P, Caci H, Young S, Kahle J, Woods AG, et al. A systematic review and analysis of long-term outcomes in attention deficit hyperactivity disorder: effects of treatment and non-treatment. BMC Med. 2012;10:99.

9. Dalsgaard S, Leckman JF, Mortensen PB, Nielsen HS, Simonsen M. Effect of drugs on the risk of injuries in children with attention deficit hyperactivity disorder: a prospective cohort study. The Lancet Psychiatry. 2015;2(8):702-9.

10. Dalsgaard S, Mortensen PB, Frydenberg M, Maibing CM, Nordentoft M, Thomsen PH. Association between Attention-Deficit Hyperactivity Disorder in childhood and schizophrenia later in adulthood. Eur Psychiat. 2014;29(4):259-63.

11. Barbaresi WJ, Colligan RC, Weaver AL, Voigt RG, Killian JM, Katusic SK. Mortality, ADHD, and Psychosocial Adversity in Adults With Childhood ADHD: A Prospective Study. Pediatrics. 2013;131(4).

12. Allely CS. The association of ADHD symptoms to self-harm behaviours: a systematic PRISMA review. BMC Psychiatry. 2014;14(1):133.

13. Chen VC-H, Chan H-L, Wu S-I, Lee M, Lu M-L, Liang H-Y, et al. Attention-Deficit/Hyperactivity Disorder and Mortality Risk in Taiwan. JAMA Network Open. 2019;2(8):e198714-e.

14. London AS, Landes SD. Attention Deficit Hyperactivity Disorder and adult mortality. Preventive medicine. 2016;90:8-10.

15. Klein B, Damiani-Taraba G, Koster A, Campbell J, Scholz C. Diagnosing attention-deficit hyperactivity disorder (ADHD) in children involved with child protection services: are current diagnostic guidelines acceptable for vulnerable populations? Child Care Health Dev. 2015;41(2):178-85.

16. Zito JM, Safer DJ, Sai D, Gardner JF, Thomas D, Coombes P, et al. Psychotropic Medication Patterns Among Youth in Foster Care. Pediatrics. 2008;121(1):e157-e63.

17. Klein RG, Mannuzza S, Olazagasti MAR, Roizen E, Hutchison JA, Lashua EC, et al. Clinical and Functional Outcome of Childhood Attention-Deficit/Hyperactivity Disorder 33 Years Later. Archives of General Psychiatry. 2012;69(12):1295-303.

18. Fletcher J. The effects of childhood ADHD on adult labor market outcomes. Cambridge, MA: National Bureau of Economic Research; 2013. Contract No.: 18689.

19. Chang Z, Lichtenstein P, Halldner L, D'Onofrio B, Serlachius E, Fazel S, et al. Stimulant ADHD medication and risk for substance abuse. Journal of Child Psychology and Psychiatry. 2014;55(8):878-85.

For peer review only - http://bmjopen.bmj.com/site/about/guidelines.xhtml

20. Dalsgaard S, Mortensen PB, Frydenberg M, Thomsen PH. ADHD, stimulant treatment in childhood and subsequent substance abuse in adulthood - A naturalistic long-term follow-up study. Addictive Behaviors. 2014;39(1):325-8.
21. Madsen AG, Dalsgaard S. Prevalence of smoking, alcohol and substance use among adolescents with attention-deficit/hyperactivity disorder in Denmark compared with the general population. Nordic Journal of Psychiatry. 2014;68(1):53-9.
22. Erskine HE, Norman RE, Ferrari AJ, Chan GC, Copeland WE, Whiteford HA, et al. Long-Term Outcomes of Attention-Deficit/Hyperactivity Disorder and Conduct Disorder: A Systematic Review and Meta-Analysis. J Am Acad Child Adolesc Psychiatry. 2016;55(10):841-50.
23. Fletcher J, Wolfe B. Long-term consequences of childhood ADHD on criminal activities. J Ment Health Policy Econ. 2009;12(3):119-38.
24. Dalsgaard S. Long-term psychiatric and criminality outcome of children with attention-deficit/hyperactivity disorder. Nordic Journal of Psychiatry. 2003;57(2):160-.
25. National Institute for Health and Care Excellence. Attention deficit hyperactivity disorder: diagnosis and management. NICE guideline [N87]2018.
26. Helsedirektoratet. ADHD/Hyperkinetisk forstyrrelse - Nasjonal faglig retningslinje for utredning, behandling og oppfølging. 2017.
27. Molina BSG, Hinshaw SP, Swanson JM, Arnold LE, Vitiello B, Jensen PS, et al. The MTA at 8 Years: Prospective Follow-up of Children Treated for Combined-Type ADHD in a Multisite Study. Journal of the American Academy of Child & Adolescent Psychiatry. 2009;48(5):484-500.
28. MTA Cooperative Group. A 14-month randomized clinical trial of treatment strategies for attention-deficit/hyperactivity disorder. The MTA Cooperative Group. Multimodal Treatment Study of Children with ADHD. Arch Gen Psychiatry. 1999;56(12):1073-86.
29. Swanson JM, Arnold LE, Molina BSG, Sibley MH, Hechtman LT, Hinshaw SP, et al. Young adult outcomes in the follow-up of the multimodal treatment study of attention-deficit/hyperactivity disorder: symptom persistence, source discrepancy, and height suppression. Journal of child psychology and psychiatry, and allied disciplines. 2017;58(6):663-78.
30. Swanson JM, Hinshaw SP, Arnold LE, Gibbons RD, Marcus SUE, Hur K, et al. Secondary Evaluations of MTA 36-Month Outcomes: Propensity Score and Growth Mixture Model Analyses. Journal of the American Academy of Child & Adolescent Psychiatry. 2007;46(8):1003-14.
31. Pliszka SR. Is There Long-Term Benefit From Stimulant Treatment for ADHD? Am J Psychiat. 2019;176(9):685-6.
32. Biederman J, Mick E, Faraone SV. Age-dependent decline of symptoms of attention deficit hyperactivity disorder: impact of remission definition and symptom type. Am J Psychiatry. 2000;157(5):816-8.
33. Hinshaw SP, Arnold LE. Attention-deficit hyperactivity disorder, multimodal treatment, and longitudinal outcome: evidence, paradox, and challenge. Wiley interdisciplinary reviews Cognitive science. 2015;6(1):39-52.
34. Storebo OJ, Krogh HB, Ramstad E, Moreira-Maia CR, Holmskov M, Skoog M, et al. Methylphenidate for attention-deficit/hyperactivity disorder in children and adolescents: Cochrane systematic review with meta-analyses and trial sequential analyses of randomised clinical trials. BMJ (Clinical research ed). 2015;351:h5203.
35. Matthijssen A-FM, Dietrich A, Bierens M, Kleine Deters R, van de Loo-Neus GHH, van den Hoofdakker BJ, et al. Continued Benefits of Methylphenidate in ADHD After 2 Years in Clinical Practice: A Randomized Placebo-Controlled Discontinuation Study. Washington, DC :2019. p. 754-62.
36. van de Loo-Neus GH, Rommelse N, Buitelaar JK. To stop or not to stop? How long should medication treatment of attention-deficit hyperactivity disorder be extended? European neuropsychopharmacology : the journal of the European College of Neuropsychopharmacology. 2011;21(8):584-99.
37. Lichtenstein P, Halldner L, Zetterqvist J, Sjolander A, Serlachius E, Fazel S, et al. Medication for attention deficit-hyperactivity disorder and criminality. The New England journal of medicine. 2012;367(21):2006-14.
38. Dalsgaard S, Mortensen PB, Frydenberg M, Thomsen PH. Long-term criminal outcome of children with attention deficit hyperactivity disorder. Criminal behaviour and mental health : CBMH. 2013;23(2):86-98.

For peer review only - http://bmjopen.bmj.com/site/about/guidelines.xhtml

39. Lundstrom S, Forsman M, Larsson H, Kerekes N, Serlachius E, Langstrom N, et al. Childhood neurodevelopmental disorders and violent criminality: a sibling control study. J Autism Dev Disord. 2014;44(11):2707-16.
40. Mohr-Jensen C, Müller Bisgaard C, Boldsen SK, Steinhausen H-C. Attention-Deficit/Hyperactivity Disorder in Childhood and Adolescence and the Risk of Crime in Young Adulthood in a Danish Nationwide Study. Journal of the American Academy of Child & Adolescent Psychiatry. 2019;58(4):443-52.
41. Quinn PD, Chang Z, Hur K, Gibbons RD, Lahey BB, Rickert ME, et al. ADHD Medication and Substance-Related Problems. Am J Psychiat. 2015;0(0):appi.ajp.2017.16060686.
42. Dalsgaard S, Nielsen HS, Simonsen M. Consequences of ADHD medication use for children's outcomes. Journal of Health Economics. 2014;37:137-51.
43. Chang Z, Lichtenstein P, Deonofrio BM, Sjolander A, Larsson H. Serious transport accidents in adults with attention-deficit/hyperactivity disorder and the effect of medication: a population-based study.(Report). JAMA Psychiatry. 2014;71(3):319.
44. Getahun D, Jacobsen SJ, Fassett MJ, Chen W, Demissie K, Rhoads GG. Recent trends in childhood attention-deficit/hyperactivity disorder. JAMA Pediatrics. 2013;167(3):282-8.
45. Atladottir HO, Gyllenberg D, Langridge A, Sandin S, Hansen SN, Leonard H, et al. The increasing prevalence of reported diagnoses of childhood psychiatric disorders: a descriptive multinational comparison. Eur Child Adolesc Psychiatry. 2015;24(2):173-83.
46. Stephenson CP, Karanges E, McGregor IS. Trends in the utilisation of psychotropic medications in Australia from 2000 to 2011. The Australian and New Zealand journal of psychiatry. 2013;47(1):74-87.
47. Man KKC, Ip P, Hsia Y, Chan EW, Chui CSL, Lam MPS, et al. ADHD Drug Prescribing Trend Is Increasing Among Children and Adolescents in Hong Kong. Journal of attention disorders. 2017;21(14):1161-8.
48. Polanczyk GV, Salum GA, Sugaya LS, Caye A, Rohde LA. Annual Research Review: A meta-analysis of the worldwide prevalence of mental disorders in children and adolescents. Journal of Child Psychology and Psychiatry. 2015;56(3):345-65.
49. Thomas R, Sanders S, Doust J, Beller E, Glasziou P. Prevalence of Attention-Deficit/Hyperactivity Disorder: A Systematic Review and Meta-analysis. Pediatrics2015. p. E994-E1001.
50. Sayal K, Prasad V, Daley D, Ford T, Coghill D. ADHD in children and young people: prevalence, care pathways, and service provision. The Lancet Psychiatry. 2017.
51. Faraone SV, Sergeant JA, Gillberg C, Biederman J, Clinical N. The Worldwide prevalence of ADHD: is it an American condition? World Psychiatry. 2003;2(2):104-13.
52. Raman SR, Man KKC, Bahmanyar S, Berard A, Bilder S, Boukhris T, et al. Trends in attention-deficit hyperactivity disorder medication use: a retrospective observational study using population-based databases. The Lancet Psychiatry. 2018.
53. Zoega H, Furu K, Halldorsson M, Thomsen PH, Sourander A, Martikainen JE. Use of ADHD drugs in the Nordic countries: a population-based comparison study. Acta Psychiatrica Scandinavica. 2011;123(5):360-7.
54. Piper BJ, Ogden CL, Simoyan OM, Chung DY, Caggiano JF, Nichols SD, et al. Trends in use of prescription stimulants in the United States and Territories, 2006 to 2016. PloS one. 2018;13(11):e0206100-e.
55. McDonald DC, Jalbert SK. Geographic variation and disparity in stimulant treatment of adults and children in the United States in 2008. Psychiatric services (Washington, DC). 2013;64(11):1079-86.
56. Fulton BD, Scheffler RM, Hinshaw SP, Levine P, Stone S, Brown TT, et al. National variation of ADHD diagnostic prevalence and medication use: health care providers and education policies. Psychiatr Serv. 2009;60(8):1075-83.
57. Madsen KB, Ersbøll AK, Olsen J, Parner E, Obel C. Geographic analysis of the variation in the incidence of ADHD in a country with free access to healthcare: a Danish cohort study. International journal of health geographics. 2015;14:24-.

For peer review only - http://bmjopen.bmj.com/site/about/guidelines.xhtml

58. Wallach-Kildemoes H, Skovgaard AM, Thielen K, Pottegard A, Mortensen LH. Social adversity and regional differences in prescribing of ADHD medication for school-age children. Journal of developmental and behavioral pediatrics : JDBP. 2015;36(5):330-41.
59. Hjern A, Weitoft GR, Lindblad F. Social adversity predicts ADHD-medication in school children--a national cohort study. Acta paediatrica (Oslo, Norway : 1992). 2010;99(6):920-4.
60. Knopf H, Holling H, Huss M, Schlack R. Prevalence, determinants and spectrum of attention-deficit hyperactivity disorder (ADHD) medication of children and adolescents in Germany: results of the German Health Interview and Examination Survey (KiGGS). BMJ Open. 2012;2(6).
61. Ørstavik R, Gustavson K, Rohrer-Baumgartner N, Biele G, Furu K, Karlstad Ø, et al. ADHD i Norge: En statusrapport. www.fhi.no: Folkehelseinstituttet; 2016.
62. Surén P, Bakken IJ, Lie KK, Schjølberg S, Aase H, Reichborn-Kjennerud T, et al. Differences across counties in the registered prevalence of autism, ADHD, epilepsy and cerebral palsy in Norway. Tidsskr Nor Laegeforen. 2013;133(18):1929-34.
63. Paul Kjetel Soldal Lillemoen SRK, Steinar Hunskår, Sabine Ruths. Prescriptions for ADHD medication, 2004 – 08. Tidsskriftet. 2012.
64. Evans WN, Morrill MS, Parente ST. Measuring inappropriate medical diagnosis and treatment in survey data: The case of ADHD among school-age children. Journal of Health Economics. 2010;29(5):657-73.
65. Karlstad O, Furu K, Stoltenberg C, Haberg SE, Bakken IJ. ADHD treatment and diagnosis in relation to children's birth month: Nationwide cohort study from Norway. Scand J Public Health. 2017;45(4):343-9.
66. Surén P, Thorstensen AG, Tørstad M, Emhjellen PE, Furu K, Biele G, et al. Diagnosis of hyperkinetic disorder among children in Norway. Tidsskr Nor Legeforen. 2018.
67. Frances A. The past, present and future of psychiatric diagnosis. World Psychiatry. 2013;12(2).
68. Lebowitz MS. Stigmatization of ADHD: A Developmental Review. J Atten Disord. 2016;20(3):199-205.
69. Lakhan SE, Kirchgessner A. Prescription stimulants in individuals with and without attention deficit hyperactivity disorder: misuse, cognitive impact, and adverse effects. Brain and behavior. 2012;2(5):661-77.
70. Kooij JJS, Bijlenga D, Salerno L, Jaeschke R, Bitter I, Balázs J, et al. Updated European Consensus Statement on diagnosis and treatment of adult ADHD. Eur Psychiat. 2019;56:14-34.
71. Elsabbagh M, Divan G, Koh YJ, Kim YS, Kauchali S, Marcin C, et al. Global prevalence of autism and other pervasive developmental disorders. Autism research : official journal of the International Society for Autism Research. 2012;5(3):160-79.
72. Collishaw S. Annual Research Review: Secular trends in child and adolescent mental health. Journal of Child Psychology and Psychiatry. 2015;56(3):370-93.
73. Frances A. Saving normal : an insider's revolt against out-of-control psychiatric diagnosis, DSM-5, Big Pharma, and the medicalization of ordinary life. New York: William Morrow; 2013.
74. Atkinson M, Hollis CJAoDiC-E, Practice. NICE guideline: attention deficit hyperactivity disorder. 2010;95(1):24-7.
75. Speerforck S, Stolzenburg S, Hertel J, Grabe HJ, Strauß M, Carta MG, et al. ADHD, stigma and continuum beliefs: A population survey on public attitudes towards children and adults with attention deficit hyperactivity disorder. Psychiatry Res. 2019;282:112570.
76. McLennan JD. Understanding attention deficit hyperactivity disorder as a continuum. Can Fam Physician. 2016;62(12):979-82.
77. Larsson H, Anckarsater H, Rastam M, Chang Z, Lichtenstein P. Childhood attention-deficit hyperactivity disorder as an extreme of a continuous trait: a quantitative genetic study of 8,500 twin pairs. Journal of child psychology and psychiatry, and allied disciplines. 2012;53(1):73-80.
78. Owens J. Relationships between an ADHD Diagnosis and Future School Behaviors among Children with Mild Behavioral Problems. 2020;0(0):0038040720909296.
79. Eccleston L, Williams J, Knowles S, Soulsby L. Adolescent experiences of living with a diagnosis of ADHD: a systematic review and thematic synthesis. Emotional and Behavioural Difficulties. 2019;24(2):119-35.

For peer review only - http://bmjopen.bmj.com/site/about/guidelines.xhtml

80. Ringer N. Living with ADHD: A Meta-Synthesis Review of Qualitative Research on Children's Experiences and Understanding of Their ADHD. International Journal of Disability, Development and Education. 2020;67(2):208-24.
81. Lebowitz MS. Stigmatization of ADHD: A Developmental Review. Journal of attention disorders. 2016;20(3):199-205.
82. Mueller AK, Fuermaier ABM, Koerts J, Tucha L. Stigma in attention deficit hyperactivity disorder. Atten Defic Hyperact Disord. 2012;4(3):101-14.
83. Brookhart MA, Schneeweiss S. Preference-based instrumental variable methods for the estimation of treatment effects: assessing validity and interpreting results. Int J Biostat. 2007;3(1):Article 14.
84. Fang G, Brooks JM, Chrischilles EA. A new method to isolate local-area practice styles in prescription use as the basis for instrumental variables in comparative effectiveness research. Med Care. 2010;48(8):710-7.
85. Ministry of Health and Care Services. Meld. St. 7 (2019-2020) National Health and Hospital Plan 2020-2023. 2019.
86. Hernán MA, Hsu J, Healy B. A Second Chance to Get Causal Inference Right: A Classification of Data Science Tasks. CHANCE. 2019;32(1):42-9.
87. Moser A, Puhan MA, Zwahlen M. The role of causal inference in health services research I: tasks in health services research. International Journal of Public Health. 2020;65(2):227-30.
88. De Montgomery CJ, Smith Jervelund S. Nordic registry data – part II: health, employment and children. 2020;48(2):123-4.
89. Smith Jervelund S, De Montgomery CJ. Nordic registry data: value, validity and future. 2020;48(1):1-4.
90. Kirkwood BR. Essential Medical Statistics. 2nd ed.. ed: Hoboken: John Wiley & Sons, Incorporated; 2003.
91. Schuster NA, Hoogendijk EO, Kok AAL, Twisk JWR, Heymans MW. Ignoring competing events in the analysis of survival data may lead to biased results: a non-mathematical illustration of competing risk analysis. Journal of Clinical Epidemiology. 2020.
92. Herle M, Micali N, Abdulkadir M, Loos R, Bryant-Waugh R, Hübel C, et al. Identifying typical trajectories in longitudinal data: modelling strategies and interpretations. Eur J Epidemiol. 2020.
93. Gelman A, Hill J. Data analysis using regression and multilevel/hierarchical models. Cambridge: Cambridge University Press; 2007.
94. Murnane RJ, Willett JB. Methods matter: Improving causal inference in educational research. Oxford, New York: Oxford University Press; 2010.
95. Stukel TA, Fisher ES, Wennberg DE, Alter DA, Gottlieb DJ, Vermeulen MJ. Analysis of observational studies in the presence of treatment selection bias: effects of invasive cardiac management on AMI survival using propensity score and instrumental variable methods. Jama. 2007;297(3):278-85.
96. McClellan M, McNeil BJ, Newhouse JP. Does more intensive treatment of acute myocardial infarction in the elderly reduce mortality? Analysis using instrumental variables. Jama. 1994;272(11):859-66.
97. Cook TD, Shadish WR, Wong VC. Three conditions under which experiments and observational studies produce comparable causal estimates: New findings from within-study comparisons. 2008;27(4):724-50.
98. Chorniy A, Kitashima L. Sex, drugs, and ADHD: The effects of ADHD pharmacological treatment on teens' risky behaviors. Labour Economics. 2016;43(Supplement C):87-105.
99. Potter BJ, Dormuth C, Le Lorier J. A theoretical exploration of therapeutic monomania as a physician-based instrumental variable. Pharmacoepidemiology and drug safety. 2019.
100. Rassen JA, Brookhart MA, Glynn RJ, Mittleman MA, Schneeweiss S. Instrumental variables II: instrumental variable application—in 25 variations, the physician prescribing preference generally was strong and reduced covariate imbalance. Journal of Clinical Epidemiology. 2009;62(12):1233-41.
101. Ionescu-Ittu R, Abrahamowicz M, Pilote L. Treatment effect estimates varied depending on the definition of the provider prescribing preference-based instrumental variables. Journal of Clinical Epidemiology. 2012;65(2):155-62.

For peer review only - http://bmjopen.bmj.com/site/about/guidelines.xhtml

102. Brooks JM, Tang Y, Chapman CG, Cook EA, Chrischilles EA. What is the effect of area size when using local area practice style as an instrument? Journal of Clinical Epidemiology. 2013;66(8, Supplement):S69-S83.
103. Kitashima L, Chorniy A. ADHD Medication Effects on Primary and Secondary Student's Academic Achievement. ADHD Medication on Child Welfare. PhD. Clemson University, South Carolina, USA: Clemson University; 2017.
104. Fallesen P, Wildeman C. The Effect of Medical Treatment of Attention Deficit Hyperactivity Disorder (ADHD) on Foster Care Caseloads:Evidence from Danish Registry Data. 2015;56(3):398-414.
105. Franklin JM, Schneeweiss S, Huybrechts KF, Glynn RJ. Evaluating possible confounding by prescriber in comparative effectiveness research. Epidemiology. 2015;26(2):238-41.
106. Dunning T. Natural experiments in the social sciences : a design-based approach. Cambridge: Cambridge University Press; 2012.
107. Magnussen J, Kalseth J. Geographical distribution of resources to health services. In: Haug K, Kaarbøe OM, Olsen TE, editors. A health service without boundaries?2009.
108. Chorniy A, Kitashima L. Sex, drugs, and ADHD: The effects of ADHD pharmacological treatment on teens' risky behaviors. Labour Economics. 2016;43:87-105.
109. Hernán M, Robins JM. Causal Inference: What if? Boca Raton: Chapman & Hall/CRC; 2020.
110. Matthay EC, Hagan E, Gottlieb LM, Tan ML, Vlahov D, Adler NE, et al. Alternative causal inference methods in population health research: Evaluating tradeoffs and triangulating evidence. SSM - Population Health. 2020;10:100526.
111. StataCorp. Stata Statistical Software: Release 16.1. College Station, TX: StataCorp LLC; 2020.
112. R Core Team. R: A language and environment for statistical computing. Vienna, Austria: R Foundation for Statistical Computing; 2020.
113. Thygesen L, Ersbøll A. When the entire population is the sample: strengths and limitations in register-based epidemiology. Eur J Epidemiol. 2014;29(8):551-8.
114. Bakken IJ, Ariansen AMS, Knudsen GP, Johansen KI, Vollset SE. The Norwegian Patient Registry and the Norwegian Registry for Primary Health Care: Research potential of two nationwide health-care registries. Scand J Public Health. 2019;48(1):49-55.

Authors' contributions: AM proposed the idea and developed it in collaboration with TW and KR. AM is PI for the project and responsible for ethics, funding, main supervision, and data management. TW, IL, and AC are PhD candidates, TW with a dual role also as statistician. FE, PB, SM and HDZ are involved for their expertise on statistics, epidemiology, and IV-methods, and have all advised on this project. FE developed the IV-strategy in collaboration with co-authors. IB and AH advised on the clinical substance matter and on the organization of ADHD treatment delivery in Norway. KR is head of the unit where the study belongs. AM planned the structure of this protocol paper, and the first draft was written in close collaboration between TW, AC, and IL. All other collaborators have contributed to the intellectual content of the paper, edited, and approved the final manuscript.

For peer review only - http://bmjopen.bmj.com/site/about/guidelines.xhtml

Funding: The project is funded by the Research Council of Norway (RCN) under the FRIMEDBIO (project number 288585) for four years starting 01.11.2019, a grant from The Western Norway Regional Health Authority from 01.01.2018 to 31.12.2023, and funding from Haukeland University Hospital. These are governmental funding bodies and the project has undergone peer-review as part of the funding application processes. The funders had no role in study design, data collection and analysis, decision to publish, or preparation of the manuscript. The project is owned by Center for Research and Education in Forensic Psychiatry, Haukeland University Hospital, Bergen, Norway, where the PI and PhD candidates on the project are currently employed.

Competing interests: None.

Acknowledgements: Several academic colleagues have advised on the development of the empirical and theoretical approach for this project in the context of the broader project "Current Controversies in Psychiatry". We acknowledge (in alphabetical order) Stål Bjørkly, Centre for Research and Education in Forensic Psychiatry, Oslo University Hospital, Oslo, Norway; Beate Brinchmann, Nordland Hospital Trust, Centre for Work and Mental Health, Bodø, Norway; Ian Colman, University of Ottawa, Canada; Sarah Dorrington, Institute of Psychiatry, Psychology and Neuroscience, King's College London, England; Kevin Douglas, Department of Psychology, Simon Fraser University, Canada; Simon Gilbody, University of York, York, England; Rolf Gjestad, Center for Research and Education in Forensic Psychiatry, Haukeland University Hospital, Bergen, Norway; Nicholas Glozier, Brain and Mind Research Institute, University of Sydney, Sydney, Australia; Stephen Hart, Department of Psychology, Simon Fraser University, Canada; Samuel Harvey, School of Psychiatry, University of New South Wales, Sydney, Australia; Hallvard Lund-Heimark, NORMENT Centre of Excellence, Haukeland University Hospital, Bergen, Norway; Matthew Hotopf, Institute of Psychiatry, Psychology and Neuroscience, King's College London, England; Erik Johnsen, Division of

For peer review only - http://bmjopen.bmj.com/site/about/guidelines.xhtml

Mental Health and NORMENT Centre of Excellence, Haukeland University Hospital, Bergen, Norway; Eoin Killackey, Orygen & Centre for Youth Mental Health, University of Melbourne, Australia; Caroline Logan, National Health Service, England; Liv Mellesdal, Division of Mental Health, Haukeland University Hospital, Bergen, Norway; Tom Palmstierna, Department of Clinical Neuroscience, Karolinska Institute, Stockholm, Sweden; Mette Senneseth, Center for Research and Education in Forensic Psychiatry, Haukeland University Hospital, Bergen, Norway; Rob Stewart, Institute of Psychiatry, Psychology and Neuroscience, King's College London, England; for valuable advice and contributions in the process of developing this project in the broader framework "Current controversies in psychiatry".

For peer review only - http://bmjopen.bmj.com/site/about/guidelines.xhtml

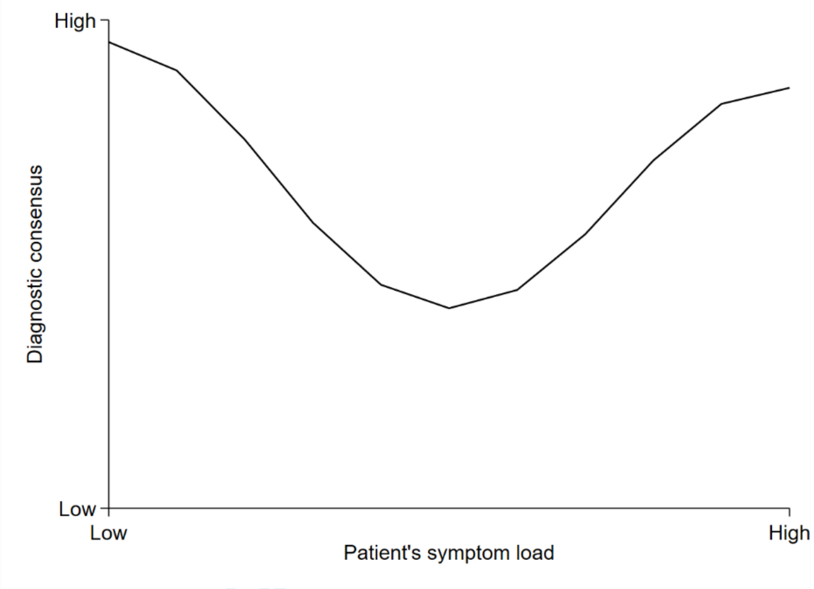

Figure 1. Theoretical model on clinician's consensus on diagnosis and medication by ADHD symptom load among referred patients.

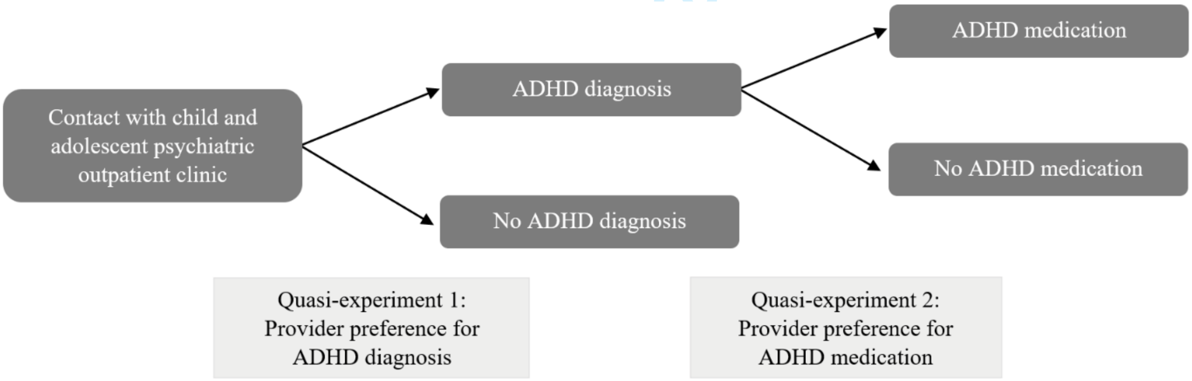

Figure 2. Two sources of as-if randomization. Quasi-experiment 1 is provider preference for ADHD diagnosis. Quasi-experiment 2 is provider preference for ADHD medication among those diagnosed with ADHD.

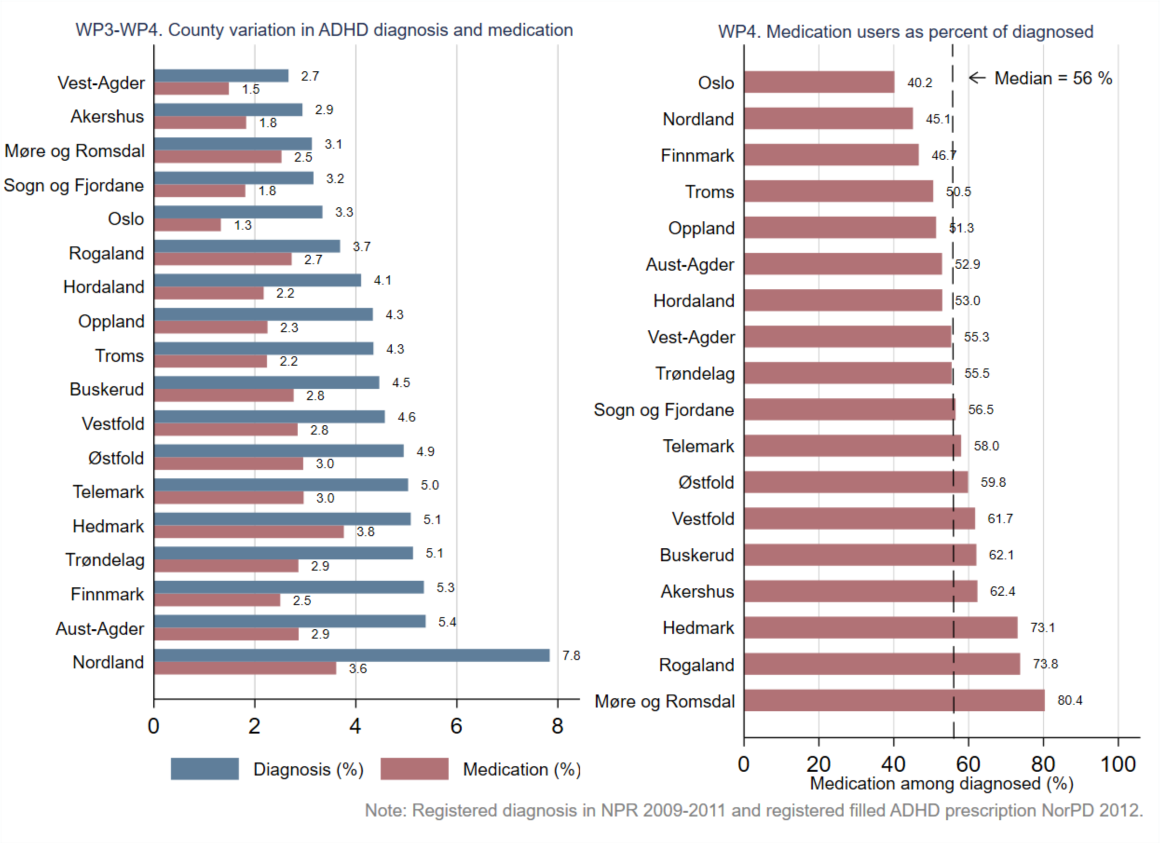

Figure 3. County variation in ADHD diagnosis and medication percent for children and adolescents between 10 to 19 years in Norway. ADHD as main diagnosis once or more times during 2009-2011.

review only

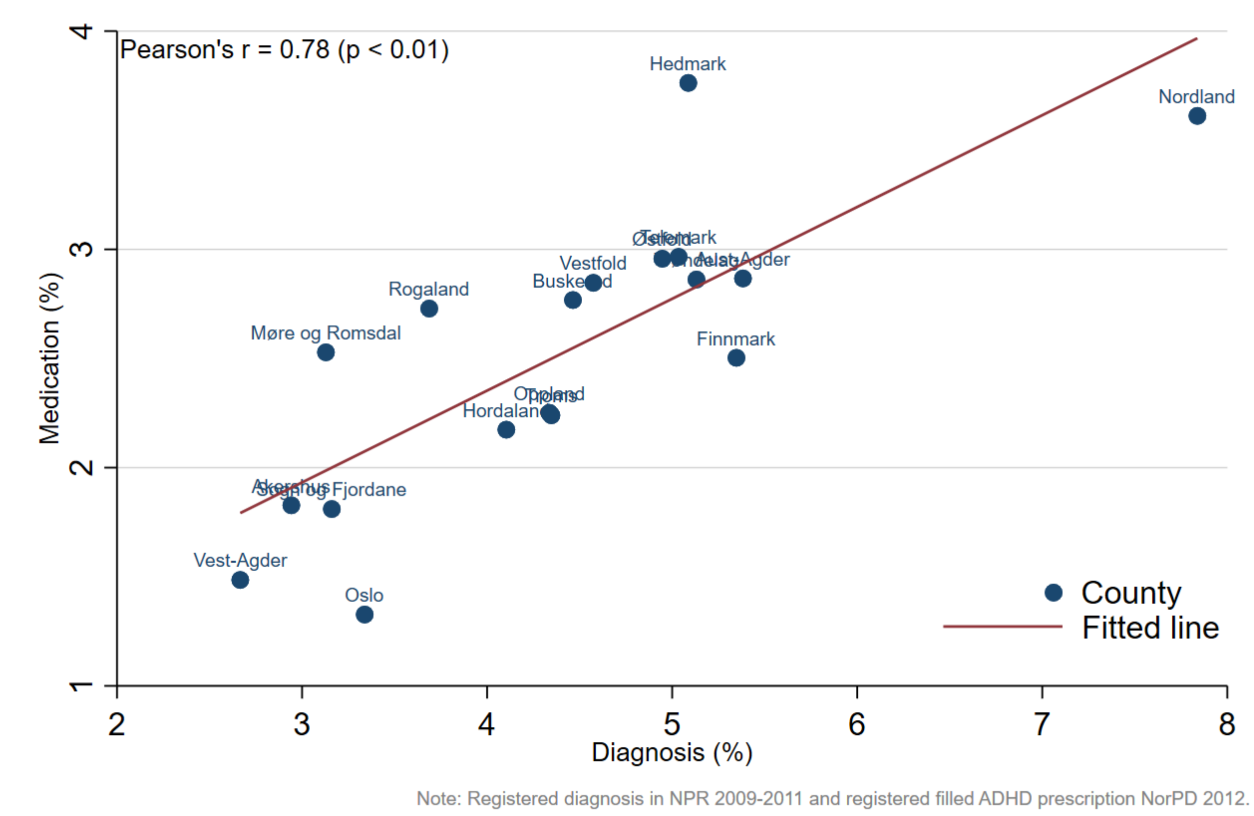

Figure 4. Scatterplot of ADHD diagnosis and medication among children and adolescents between 10 to 19 years by Norwegian counties. ADHD as main diagnosis once or more times during 2009-2011. Linear fitted line.

For peer review only

SPIRIT

STANDARD PROTOCOL ITEMS: RECOMMENDATIONS FOR INTERVENTIONAL TRIALS

SPIRIT 2013 Checklist: Recommended items to address in a clinical trial protocol and related documents*

Section/item	Item No	Description
**Administrative information**		
Title	1	Page 1
Trial registration	2a	Submitted to ISRCTN trial registry (pg. 4)
Protocol version	3	N/A
Funding	4	Funding (pg. 29)
Roles and responsibilities	5a	Page 1, Author's contribution (pg 28).
	5b	Funding, (pg. 29). Contact information: Arnstein Mykletun, Principle Investigator, email: arnstein.mykletun@uit.no (pg.2)
	5c	Funding (pg. 29).
	5d	N/A
**Introduction**		
Background and rationale	6a	Introduction (pg. 5-9).
	6b	Data and study sample (pg. 15-16) and WP3 and WP4: Long-term effects of receiving ADHD diagnosis and ADHD medication (pg. 11-12).
Objectives	7	Introduction, paragraph 12-16 (pg.9-10).
Trial design	8	N/A
**Methods: Participants, interventions, and outcomes**		
Study setting	9	Data and study sample, paragraph 1 (pg. 15-16).

For peer review only - http://bmjopen.bmj.com/site/about/guidelines.xhtml

Eligibility criteria	10	Data and study sample, paragraph 1 (pg. 15-16).
Interventions	11a	N/A
	11b	N/A
	11c	N/A
	11d	N/A
Outcomes	12	Data and study sample, paragraph 2 (pg. 16).
Participant timeline	13	Data and study sample, paragraph 1,2 (pg. 15-16).
Sample size	14	Data and study sample, paragraph 2 (pg. 16).
Recruitment	15	Data and study sample, paragraph 2 (pg. 16), Table 1 (pg. 16-17).

Methods: Assignment of interventions (for controlled trials) (N/A)

Allocation:		
Sequence generation	16a	N/A.
Allocation concealment mechanism	16b	N/A.
Implementation	16c	N/A
Blinding (masking)	17a	N/A
	17b	N/A

Methods: Data collection, management, and analysis

Data collection methods	18a	Data and study sample, paragraph 2-3 (pg.16), Table 1 (pg. 16-17).
	18b	N/A
Data management	19	Data management (pg.20).
Statistical methods	20a	Methods, paragraph 2-8 (pg. 11-15).
	20b	N/A
	20c	Data management (pg. 20).

Methods: Monitoring

Data monitoring	21a	N/A. DMC not needed as we are using register data that are already collected.
	21b	N/A
Harms	22	N/A
Auditing	23	N/A
**Ethics and dissemination**		
Research ethics approval	24	Ethics approval and consent to participate (pg. 20).
Protocol amendments	25	Dissemination, paragraph 7 (pg. 22).
Consent or assent	26a	N/A as the study is based on registry data. See Ethical approval and consent to participate (pg. 20).
	26b	N/A
Confidentiality	27	Data management (pg. 20), Ethical approval and consent to participate (pg. 20), Availability of data and materials (pg. 20-21)
Declaration of interests	28	Pg. 20,28-29.
Access to data	29	Availability of data and materials (pg. 21)
Ancillary and post-trial care	30	N/A
Dissemination policy	31a	Dissemination, paragraph 1-7 (pg. 21-22).
	31b	Dissemination, paragraph 7 (pg. 22).
	31c	Dissemination, paragraph 7 (pg.22), Availability of data and materials (pg.20-21)
**Appendices**		
Informed consent materials	32	N/A as we are using registry-based data.
Biological specimens	33	N/A

*It is strongly recommended that this checklist be read in conjunction with the SPIRIT 2013 Explanation & Elaboration for important clarification on the items. Amendments to the protocol should be tracked and dated. The SPIRIT checklist is copyrighted by the SPIRIT Group under the Creative Commons "Attribution-NonCommercial-NoDerivs 3.0 Unported" license.

For peer review only

BMJ Open

Causal Modeling of Variation in Clinical Practice and Long-Term Outcomes of ADHD using Norwegian Registry Data: The ADHD Controversy Project

Journal:	*BMJ Open*
Manuscript ID	bmjopen-2020-041698.R1
Article Type:	Protocol
Date Submitted by the Author:	30-Nov-2020
Complete List of Authors:	Mykletun, Arnstein; University of Tromso Faculty of Health Sciences, Department of Community Medicine; Haukeland University Hospital, Centre for Research and Education in Forensic Psychiatry<br>Widding-Havneraas, Tarjei; Haukeland University Hospital, Centre for Research and Education in Forensic Psychiatry; University of Bergen, Department of Clinical Medicine<br>Chaulagain, Ashmita; Haukeland University Hospital, Centre for Research and Education in Forensic Psychiatry; University of Bergen, Department of Clinical Medicine<br>Lyhmann, Ingvild; Haukeland University Hospital, Centre for Research and Education in Forensic Psychiatry; University of Bergen, Department of Clinical Medicine<br>Bjelland, Ingvar; University of Bergen, Department of Clinical Medicine; Haukeland University Hospital, Department of Psychiatry<br>Halmøy, Anne; University of Bergen, Department of Clinical Medicine; Haukeland University Hospital, Department of Psychiatry<br>Elwert, Felix; University of Wisconsin-Madison, Department of Sociology<br>Butterworth, Peter; University of Melbourne<br>Markussen, Simen; Ragnar Frisch Centre for Economic Research,<br>Zachrisson, Henrik; University of Oslo, Department of Special Education<br>Rypdal, Knut; Haukeland University Hospital, Centre for Research and Education in Forensic Psychiatry
**Primary Subject Heading**:	Epidemiology
Secondary Subject Heading:	Health services research, Mental health
Keywords:	Child & adolescent psychiatry < PSYCHIATRY, MENTAL HEALTH, EPIDEMIOLOGY

For peer review only - http://bmjopen.bmj.com/site/about/guidelines.xhtml

I, the Submitting Author has the right to grant and does grant on behalf of all authors of the Work (as defined in the below author licence), an exclusive licence and/or a non-exclusive licence for contributions from authors who are: i) UK Crown employees; ii) where BMJ has agreed a CC-BY licence shall apply, and/or iii) in accordance with the terms applicable for US Federal Government officers or employees acting as part of their official duties; on a worldwide, perpetual, irrevocable, royalty-free basis to BMJ Publishing Group Ltd ("BMJ") its licensees and where the relevant Journal is co-owned by BMJ to the co-owners of the Journal, to publish the Work in this journal and any other BMJ products and to exploit all rights, as set out in our licence.

The Submitting Author accepts and understands that any supply made under these terms is made by BMJ to the Submitting Author unless you are acting as an employee on behalf of your employer or a postgraduate student of an affiliated institution which is paying any applicable article publishing charge ("APC") for Open Access articles. Where the Submitting Author wishes to make the Work available on an Open Access basis (and intends to pay the relevant APC), the terms of reuse of such Open Access shall be governed by a Creative Commons licence – details of these licences and which Creative Commons licence will apply to this Work are set out in our licence referred to above.

Other than as permitted in any relevant BMJ Author's Self Archiving Policies, I confirm this Work has not been accepted for publication elsewhere, is not being considered for publication elsewhere and does not duplicate material already published. I confirm all authors consent to publication of this Work and authorise the granting of this licence.

For peer review only

Causal Modeling of Variation in Clinical Practice and Long-Term Outcomes of ADHD using Norwegian Registry Data: The ADHD Controversy Project

For peer review only

Arnstein Mykletun[1,2,3,4], Tarjei Widding-Havneraas[2,5], Ashmita Chaulagain[2,5], Ingvild Lyhmann[2,5], Ingvar Bjelland[5,6], Anne Halmøy[5,6], Felix Elwert[7], Peter Butterworth[8,9], Simen Markussen[10], Henrik Daae Zachrisson[11], Knut Rypdal[2]

[1] UiT - Department of Community Medicine, The Arctic University of Norway, Tromsø, Norway

[2] Centre for Research and Education in Forensic Psychiatry, Haukeland University Hospital, Bergen, Norway

[3] Norwegian Institute of Public Health, Oslo, Norway

[4] Nordland Hospital Trust, Centre for Work and Mental Health, Bodø, Norway

[5] Department of Clinical Medicine, University of Bergen, Norway

[6] Department of Psychiatry, Haukeland University Hospital, Bergen, Norway

[7] Department of Sociology, University of Wisconsin-Madison, WI, USA

For peer review only - http://bmjopen.bmj.com/site/about/guidelines.xhtml

[8] Research School of Population Health, Australian National University, Canberra, Australia

[9] Melbourne Institute of Applied Economic and Social Research, University of Melbourne, Melbourne, Australia

[10] Ragnar Frisch Centre for Economic Research, Oslo, Norway

[11] Department for Special Education, University of Oslo, Oslo, Norway

Corresponding author: Arnstein Mykletun: arnstein.mykletun@uit.no

Tarjei Widding-Havneraas: tarjei.widding-havneraas@helse-bergen.no

Ashmita Chaulagain: ashmita.chaulagain@helse-bergen.no

Ingvild Lyhmann: ingvild.lyhmann@helse-bergen.no

Ingvar Bjelland: ingvar.bjelland@uib.no

Anne Halmøy: anne.halmoy@uib.no

Felix Elwert: elwert@wisc.edu

Peter Butterworth: peter.butterworth@unimelb.edu.au

Simen Markussen: simen.markussen@frisch.uio.no

Henrik Daae Zachrisson: h.d.zachrisson@isp.uio.no

Knut Rypdal: knut.rypdal@helse-bergen.no

For peer review only

Word count: 6812 (excluding abstract and references).

Word count: 6118 (excluding abstract, tables, references, and acknowledgements).

Abstract

Introduction: Attention-Deficit/Hyperactivity Disorder (ADHD) is among the most common mental disorders in children and adolescents, and it is a strong risk factor for several adverse psychosocial outcomes over the lifespan. There are large between- and within-country variations in diagnosis and medication rates. Due to ethical and practical considerations, few studies have examined the effects of receiving a diagnosis, and there is a lack of research on effects of medication on long-term outcomes.

Our project has four aims organized in four work packages: (WP1) To examine the prognosis of ADHD (with and without medication) compared to patients with other psychiatric diagnoses, patients in contact with public sector child and adolescent psychiatric outpatient clinics (without diagnosis), and the general population; (WP2) Examine within-country variation in ADHD diagnoses and medication rates by clinics' catchment area; (WP3) Identify causal effects of being diagnosed with ADHD and (WP4) ADHD medication on long-term outcomes.

Method and analysis: Our project links several nationwide Norwegian registries. The patient sample is all persons aged 5-18 years that were in contact with public sector child and adolescent psychiatric outpatient clinics in 2009-2011. Our comparative analysis of prognosis will be based on survival analysis and mixed-effects models. Our analysis of variation will apply mixed-effects models and generalized linear models. We have two identification strategies for the effect of being diagnosed with ADHD and of receiving medication on long-term outcomes. Both strategies rely on using preference-based instrumental variables, which in our project are based on provider preferences for ADHD diagnosis and medication.

Ethics and dissemination: The project is approved by the Regional Ethics Committee, Norway (REC number 2017/2150/REC south-east D). All papers will be published in open access journals and results will be presented in national and international conferences.

For peer review only - http://bmjopen.bmj.com/site/about/guidelines.xhtml

Trial registration: ISRCTN11573246 and ISRCTN11891971

This is an open access article distributed in accordance with the Creative Commons Attribution Non Commercial (CC BY-NC 4.0) license, which permits others to distribute, remix, adapt, build upon this work non-commercially, and license their derivative works on different terms, provided the original work is properly cited, appropriate credit is given, any changes made indicated, and the use is non-commercial. See: http://creativecommons.org/licenses/by-nc/4.0/.

Strengths and limitations of this study:

- This study will use reliable and complete national registry data to study real-world effects of ADHD diagnosis and medication on long-term psychosocial outcomes highly relevant for patients, families and society at large.
- We utilize observed variations in rates of ADHD diagnoses and medication between public sector child and adolescent psychiatric outpatient clinics for causal modelling, enabling empirical investigation of research questions which cannot be addressed in conventional RCTs for ethical and practical reasons.
- In Norway, ADHD in children and adolescents is almost exclusively diagnosed in public sector specialist services, which is covered by our registry data, ensuring representative data for the Norwegian general population.
- The observed variation in ADHD diagnosis and medication within Norway reflects most of the variation observed between countries, relevant to the external validity of the study.
- Whereas the strength of registry data as used in this study is related to data completeness and reliability, the weakness relates to validity and in some cases also data availability.

For peer review only - http://bmjopen.bmj.com/site/about/guidelines.xhtml

Introduction

Attention-Deficit/Hyperactivity Disorder (ADHD) is among the most common mental disorders in children and adolescents (1-4), and is often associated with problems into adulthood (5). ADHD is a persistent neurodevelopmental disorder characterized by symptoms of inattention and/or hyperactivity and impulsivity that are excessive for an individual's age or overall development (6, 7). ADHD is a strong risk factor for school dropout (8), injuries (9), comorbidity (10), self-harm and mortality (11-14), contact with child welfare services (15, 16), as well as lack of employment (17, 18), later substance use disorders (17, 19-21) and criminality (22-24).

Clinical treatment guidelines recommend a multimodal approach that combine psychosocial and pharmacological treatment options (25, 26). Behavioral intervention such as parent training is presently considered an important part of the treatment for patients with ADHD (27). While not proven to be effective in reducing core ADHD symptoms, evidence shows it has positive effects on parenting and reduces oppositional and noncompliant behaviors in children, and may also lead to improvement in parent-child relationships as well as children's emotional, social and academic functioning.

In the case of pharmacological treatment, on the other hand, several clinical trials have provided evidence of psychostimulants' safety and efficacy in reducing ADHD symptoms in the short-term (28, 29) . Short-term studies show that medication reduces symptoms in at least 75 % of children and adolescents with ADHD (30), suggesting that medication may be superior to both psychological interventions and no treatment (31). Evidence from a recent systematic review and network meta-analysis of 133 double-blind randomized controlled trials concluded that the first-choice medications for the short-term treatment of ADHD should be methylphenidate and amphetamines for children/adolescents and adults, respectively (29) .

For peer review only - http://bmjopen.bmj.com/site/about/guidelines.xhtml

While short-term effects of medication are established (although with some discussions surrounding the evidence base) (32), there are few studies on long-term effects of such treatment (29). Further, what general conclusion to draw from the existing findings on this topic is still under discussion, as illustrated by a recent debate section in *Journal of the American Academy of Child & Adolescent Psychiatry* (33-35). This debate illuminated that although several long-term studies indicate positive consequences of ADHD medication use, effect sizes are typically reduced compared to short-term results; additionally, in an even longer time perspective, it seems that the effectiveness of treatment-as-usual wanes and may not even be significant (35). From the opposing standpoint of the debate, however, it is proposed that the apparent lack of long-term benefits of medication is likely caused by insufficient monitoring and adjustment of treatment for patients followed up over long periods of time, and should not be used as a general argument against the effectiveness of medication in the treatment of ADHD (34).

A promising source of long-term effect estimates to contribute to this debate is nationwide register studies (5, 36, 37). In recent years, register studies using quasi-experimental designs have shown that medication may reduce risk of unwanted outcomes associated with ADHD such as crime (38-41), substance abuse (42), injuries (9, 43), and motor vehicle accidents (44). However, as summarized in a recent review covering register based studies on the effects of ADHD medication on a wide range of behavioral and neuropsychiatric outcomes, the available evidence even from register based pharmacoepidemiological studies on long-term effects of ADHD medication are still less clear than studies on short-term effects, and are subject to several limitations (37). As pointed out by these authors and others, more long-term studies that examine functional outcomes in real-world settings are needed (e.g., 28, 29, 34, 37, 45).

Over recent decades there have been substantial increases in clinical diagnosis and pharmacological treatment of ADHD in many countries (2), including North America (46),

For peer review only - http://bmjopen.bmj.com/site/about/guidelines.xhtml

Europe (47), Australia (48), and Asia (49). Recent systematic reviews and meta-analyses estimate that the global prevalence rate of ADHD in children and adolescents is between 3.4 % (50) and 7.2 % (51). It has been estimated that at least an additional 5 % of children and adolescents have substantial difficulties with excessive and impairing levels of over-activity, inattention and impulsivity that are just below the threshold for diagnostic criteria (52). Some studies attribute the variation in prevalence estimates to social and cultural factors (53), while others downplay geographical location and argue for a methodological explanation (3).

A retrospective study based on population-level databases from 13 countries and 154.5 million individuals showed that prevalence of ADHD medication use in children aged 3-18 years varied between 0.27 % and 6.69 % across included countries (54). Among the Nordic countries, the prescription prevalence has been estimated to range from 0.12 % in Finland to 1.25 % in Iceland (55). Further, large within-country variation in prescription rates has been reported in several countries, e.g. the USA (56-58), Denmark (59, 60), Sweden (61), and Germany (62).

Even within Norway, a country with a modest inequality and a universal healthcare system, there are substantial regional differences in diagnosis and medication (63, 64). For instance, the county-wise rates for ADHD diagnosis in Norway at ages 6-12 vary between 1.7 % to 4.8 % for boys and between 0.4 % to 2.0 % for girls, and similar variation has been noted for medication rates (63). In total, 80 % of the children and adolescents diagnosed with ADHD fill one prescription for ADHD medication (63), and approximately 50 % continue to collect prescriptions for more than one year (65). In addition to geographical variation in diagnosis and medication rates, studies have shown that being young relative to peers is a strong predictor for being diagnosed with ADHD in several countries (66, 67). A Norwegian chart review indicated that only 49 % of all diagnoses were adequately documented (68).

For peer review only

Internationally, the variation in ADHD diagnosis and medication rates have caused a debate with two opposing perspectives. On the one hand, there is a restrictive perspective characterized by concerns about potential overdiagnosis of ADHD, leading to medicalization of normal behavior (69), unnecessary stigma (70), and side-effects of pharmacological treatment (71) that might not even be helpful in the presence of true ADHD. On the other hand, there is a liberal perspective distinguished by concerns about underdiagnosis and undertreatment (72), which argues that increased rates of ADHD diagnosis and medication are due to improved recognition and understanding by professionals (73, 74), broadened diagnostic definitions (75), and methodological problems in the literature (2). This perspective emphasizes the importance of reducing the risk of adverse outcomes associated with untreated ADHD, such as injuries (9), school dropout (8), and criminal behavior (43).

This debate may be reflected in clinical practice, where patients referred to specialist healthcare with suspicion of ADHD present with various degrees and compositions of symptoms (Figure 1). Clinicians generally regard ADHD symptoms on a continuum. Although the diagnostic manuals define a symptomatic and functional cut-off for ADHD (76), diagnosis is inevitably based on a clinical judgement. This discretionary decision is likely to vary between clinicians (63), partly due to underlying restrictive or liberal perspectives. Figure 1 presents our theoretical model on clinicians' consensus on diagnosis and medication by ADHD symptom load among patients in contact with child and adolescent psychiatric outpatient clinics. Clinicians will generally agree that patients with very few or no symptoms of ADHD should receive neither a diagnosis nor medication (left side of Figure 1) (77-79). On the opposite end of the continuum are patients with obvious ADHD symptoms, for whom most if not all clinicians will agree on ADHD diagnosis and medication (right side of Figure 1).

For peer review only - http://bmjopen.bmj.com/site/about/guidelines.xhtml

Figure 1. Theoretical model on clinicians' consensus on diagnosis and medication by ADHD symptom load among referred patients.

The controversy on ADHD diagnosis and ADHD medication mostly relates to the middle area of the symptom distribution in Figure 1, which includes patients with ambiguous symptoms, bordering on the threshold for diagnostic cut-off. These patients may be presenting with symptoms compatible also with other diagnoses or could be experiencing psychosocial stress that may produce ADHD-like symptoms, making an ADHD diagnosis difficult to establish.

In light of increasing ADHD diagnosis and medication rates in most Western countries, it is important to improve our knowledge on the effect of diagnosis among patients around the clinical threshold for diagnosis, as well as the effects of medication on long-term outcomes among patients. This is an important issue to address as it can inform the debate on under- and overdiagnosis and medication. A pressing issue is whether patients with ambiguous ADHD symptoms benefit or not from being diagnosed. As pointed out by Owens (80), an ADHD diagnosis "… can bring beneficial pharmacological treatment and social supports, but it can also trigger negative social and psychological processes, as suggested by labeling theory". Being diagnosed entails a set of at least three components that interact on long-term outcomes: (i) stigma ("diagnosis labeling effects") (77, 81-84), (ii) medication, and (iii) psychosocial treatment. Owens (80) finds that consistent with labeling theory (stigma), only those with severe pre-diagnosis symptoms seem to benefit from diagnosis and medication, while those with mild pre-diagnosis symptoms have worse outcomes compared to undiagnosed matches.

The key issue in identifying an effect of receiving a diagnosis is that diagnoses cannot be randomized to patients. Hence, we must either rely on epidemiological designs that precludes causal inference unless all potential confounding is statistically controlled, or we need to find

For peer review only - http://bmjopen.bmj.com/site/about/guidelines.xhtml

sources of plausibly exogenous variation that can be used as a natural experiment. We believe the geographical variation in diagnosis and medication can be used as a natural experiment to identify causal effects of diagnosis and medication on long-term outcomes. There are several plausible explanations for geographical variation in diagnosis and medication rates on both the supply and demand side, including resources, capacity, and patient mix. Nevertheless, a portion of the variation may be due to variation in clinician's diagnosis and medication practice among public sector child and adolescent psychiatric outpatient clinics, commonly referred to as provider preference (85), which can lead to variation in diagnosis and prescription decisions for similar patients. Geographical variation may induce some random variation in treatment status for patients and may be used as an instrumental variable to obtain treatment effects (86). Considering the regional differences in ADHD diagnosis and treatment, we expect that the child and adolescent outpatient clinics in Norway have different preferences. In single-provider universal access health systems, variation in health service provision that is not accounted for by differences in patient populations and symptom load is usually regarded as unwarranted and unwanted (87).

The current research project aims to contribute to the existing knowledge on ADHD diagnosis and ADHD medication in several ways. Our project will employ Norwegian registry data to explore four main aims with corresponding work packages:

WP1: The epidemiology of ADHD prognosis. The main aim of this WP is to describe the general long-term prognosis of ADHD compared to the general population. This is in response to the typical parental concern: "My child is diagnosed with ADHD, how will he/she do in life?" By long-term, we here mean years rather than months, including the important transition from childhood and adolescence to early adult life. We will examine the prognosis of patients diagnosed with ADHD in child and adolescent psychiatry (with and without medication). These will be compared to patients in child and adolescent psychiatry who received other diagnoses,

For peer review only

or who received no diagnosis. We will also compare to the general population without contact with child and adolescent psychiatry. Measures of prognosis include registry data records of emergency care visits/injuries, comorbidity, contact with child welfare services, contact with adult psychiatric health services, education, employment and income, welfare dependency, crime, self-harm, suicide, and mortality.

WP2: The ADHD variation controversy. This WP has two aims: first, we will describe the geographical variation in ADHD diagnosis and medication in Norway. We will describe this variation at the level of the catchment areas for child and adolescent psychiatric outpatient clinics in Norway. We will describe the variation in ADHD diagnosis as proportion of children and adolescents referred to these clinics and as proportion of children and adolescents in the catchment area, and also in ADHD medication as proportion of diagnosed patients. Second, we aim to explain this variation by three strategies:

a. We will explore to what extent this variation in clinical practice can be attributed to catchment area characteristics. Data on catchment areas will also be included in WP3 and WP4 as potential confounding variables.
b. Further, we will explore to what extent it can be attributed to the variation in observed ADHD symptoms as observed in a nationwide health study of the general population (88). At an aggregated level, a strong positive association between the observed symptoms (as self-reported by mothers) and rates of diagnoses and medication (as observed in registry data) will challenge the instrument planned for this study.
c. Finally, we will survey clinicians working at the about 90 child and adolescent psychiatric outpatient clinics in Norway, exploring the variation in their attitudes and opinions towards ADHD diagnosis and medication. Specifically, we will explore if these attitudes and opinions vary on a continuum from restrictive to liberal. We will also explore if this variation in attitudes and opinions correlate at the aggregated level of the

For peer review only - http://bmjopen.bmj.com/site/about/guidelines.xhtml

clinics with observed rates of ADHD diagnosis and medication (according to the registry data), and with the variation in ADHD symptoms (according to the health study (88)).

WP3: Long-term effects of receiving an ADHD diagnosis. The main aim of this WP is to examine if it makes any long-term difference for the prognosis of patients if they attend clinics with liberal or a restrictive practice on ADHD diagnosing. We assume that clear ADHD will be diagnosed at all clinics, and that clear non-ADHD patient will not receive the ADHD-diagnosis anywhere. Consequently, this WP aims to analyze the effect of ADHD diagnosis for patients on the margin of receiving the diagnosis, who are most likely to be treated differently at clinics with a liberal versus restrictive approach to the diagnosis. We will identify causal effects of being diagnosed with ADHD among patients in contact with child and adolescent psychiatric outpatient clinics on long-term outcomes including emergency care visits/injuries, comorbidity, education, contact with child welfare services, contact with adult psychiatric health services, employment and income, welfare dependency, crime, self-harm, suicide, and mortality.

WP4: Long-term effects of ADHD medication. The main aim of this WP is to examine if it makes any long-term difference for the prognosis of patients diagnosed with ADHD whether they attend a clinic with a liberal or restrictive ADHD medication practice. Not all patients who receive the ADHD diagnosis will be prescribed medication, and we assume that patients who are on the margin of receiving such treatment will be treated differently at clinics with a liberal versus restrictive approach. We will identify causal effects of initial ADHD medication among patients diagnosed with ADHD on long-term outcomes including emergency care visits/injuries, comorbidity, education, contact with child welfare services, contact with adult psychiatric health services, employment and income, welfare dependency, crime, self-harm, suicide, and mortality.

For peer review only - http://bmjopen.bmj.com/site/about/guidelines.xhtml

Registry-based studies on effects of ADHD-medication that address limitations of RCTs, such as generalizability, follow-up time and less frequent psychosocial outcomes, is expanding (37). The main challenge of these studies is confounding, which several studies try to address by research designs such as within-subjects designs (37), difference-in-differences (9) and instrumental variables (IV) (43, 89). Our project's original contribution lies in using two identification strategies which have not previously been applied to evaluate effects of diagnosis nor medication on several included outcomes. There has only been conducted one IV analysis of ADHD medication effects on hospital contacts and criminal behavior using Danish nationwide registry data and one IV study on ADHD medication effects on teens risk of sexual behavior outcomes, substance abuse and injuries using Medicaid data (89).

Methods and analysis

Project period

The project is funded by a grant from the Western Norway Regional Health Authority starting 01.01.2018 lasting six years, and a grant from the Research Council of Norway (RCN) lasting four years starting 01.11.2019. The project period may be extended with additional funding and for personnel-related reasons.

Design

Our project employs methods for descriptive analyses and causal inference (90, 91). Our study draws on several linked Norwegian nationwide registries. Nordic registry data provide individual level data for the whole population (92), and are only censored by migration and death (93). Our project has four work packages:

WP1: The epidemiology of ADHD prognosis

This first work-package is conventional epidemiology, analyzing outcomes (prognosis) in the ADHD population compared to the general population. The aim is to provide general data on

For peer review only - http://bmjopen.bmj.com/site/about/guidelines.xhtml

the prognosis of the ADHD population, employing all available outcomes. We will use survival analysis (94) to examine time-to-event for our outcomes. In line with recent studies (95), we will account for possible competing risks to avoid overestimation of the cumulative incidence of our events of interest. We will also apply mixed-effects analysis, which is well-suited and an established standard for individual-level longitudinal data with repeated observations (94, 96).

WP2: The ADHD variation controversy

This second WP will employ conventional statistics including scatter plots and regression models. Most analyses will be performed on data aggregated to geographical regions (counties or finer) and (when data allows for this) to the about 90 child and adolescent psychiatric outpatient clinic catchment areas. To account for patient clustering and repeated observations over time, we will use standard generalized linear models with cluster-robust standard errors and mixed-effects models (94, 97).

WP3 and WP4: Long-term effects of receiving an ADHD diagnosis and ADHD medication

To identify the effect of ADHD diagnosis (WP3) and ADHD medication (WP4) on long-term outcomes, we will use child and adolescent psychiatric outpatient clinics' catchment area variation in diagnosis and medication as instrumental variables (IV). IV is a common identification strategy in health-services research (98), that exploits the random variation induced in a treatment variable (here, ADHD diagnosis or ADHD medication prescription) by a plausibly exogenous (as-if random) event or process (i.e., the instrument). The role of the instrument is to "isolate" exogenous variation in the treatment, and hence to remove the endogenous (confounded) variation, so that the analysis only uses the exogenous variation to estimate the treatment effect (99). In this sense, IV analysis is a quasi-experimental approach. There is evidence to support that research designs with valid IVs can provide similar estimates as randomized experiments on the same research questions (100-102), whereas more traditional observational studies without a credible source of exogenous variation often do not (99, 102).

For peer review only - http://bmjopen.bmj.com/site/about/guidelines.xhtml

For peer review only

Our identification strategies exploit as-if random variation in providers' preference to diagnose patients with ADHD and to prescribe ADHD medication (43, 89) across Norwegian public sector child and adolescent psychiatric outpatient clinics. The main idea behind provider preference-based instruments is that "…different providers or groups of providers have different preferences dictating how medications or medical procedures are used." (85). If the provider preference is associated with treatment, does not directly affect the outcome, and is not associated with unmeasured risk factors of the outcome, it may be used as an IV to consistently estimate the treatment effect (86). We will use two candidate provider preference instruments, illustrated in Figure 2.

Figure 2. Two sources of as-if randomization. Quasi-experiment 1 is provider preference for ADHD diagnosis. Quasi-experiment 2 is provider preference for ADHD medication among those diagnosed with ADHD.

Preference-based instrumental variables

It is plausible that a provider's preference for diagnosing ADHD affects whether or not an individual is diagnosed with ADHD, while it is not associated with the outcomes of interest (i.e. via paths other than affecting diagnosis) net of certain controls. We will use provider preference for ADHD diagnosis to identify the effect of diagnosis on long-term outcomes. Similarly, provider preference for ADHD medication likely affects whether or not a patient receives medication, while it is not associated with our outcomes of interest, net of certain controls. Provider preference for ADHD medication will be used to identify the effect of ADHD medication on long-term outcomes. Provider preferences for medication have become a well-established IV in epidemiology (103), as they are usually strongly associated with medication

For peer review only - http://bmjopen.bmj.com/site/about/guidelines.xhtml

use and reduce covariate imbalance (104). Our IVs will be defined at the provider-level (i.e. the catchment area of child and adolescent psychiatric outpatient clinics), which may be stronger and more efficient compared to physician-level instruments (105). The (catchment) area definition of child and adolescent psychiatric outpatient clinics strikes a reasonable balance between methodological issues introduced by too large or too small area definitions (106).

To our knowledge, provider-level preference for diagnosis (63, 64) is a novel candidate instrument, while provider preference for ADHD medication has only been used in at least four prior studies (43, 89, 107, 108), which do not cover all outcomes in our project. We illustrate the variation that will be used as provider preference instruments for the effects of diagnosis (WP3) and medication (WP4) at the county level in Figure 3. Our eventual analysis will use preferences measured at the level of catchment areas of child and adolescent psychiatric outpatient clinics.

Figure 3. County variation in ADHD diagnosis and medication percent for children and adolescents between 10 to 19 years in Norway. ADHD as main diagnosis registered once or more times during 2009-2011.

Although rates of diagnosis and medication follow each other closely, preliminary evidence on the county level indicates that they do not correlate perfectly, as shown in Figure 3 and Figure 4.

For peer review only - http://bmjopen.bmj.com/site/about/guidelines.xhtml

Figure 4. Scatterplot of ADHD diagnosis and medication among children and adolescents between 10 to 19 years by Norwegian counties. ADHD as main diagnosis registered once or more times during 2009-2011. Linear fitted line.

Setting of study and assessment of identification strategy

Preference-based instruments may lead to biased inferences if the identifying assumptions are not met (109). The application of IV rests on a set of key assumptions that must be met to warrant a credible causal interpretation of estimates. Overall, an IV-strategy can be evaluated on a continuum from low to high credibility based on the underlying assumptions (110). The two main assumptions of IV are relevance and exclusion. Relevance can be tested statistically, while exclusion can only be partially tested and relies on justification derived from subject-matter knowledge. The instruments are likely to meet the relevance assumptions as public sector child and adolescent psychiatric outpatient clinics are the only institutions in Norway that can diagnose ADHD and initiate pharmacological treatment of ADHD (aside from a small number of private practices), and the share of patients diagnosed with ADHD and medicated for ADHD varies considerably across areas.

We expect that the instrument meets the exclusion restriction in several respects. We expect provider preference for diagnosis and medication only to affect long-term outcomes through diagnosis and treatment decisions. Although patients in Norway are free to choose outpatient clinics, which opens the door to self-selection bias in principle, in practice, the vast majority of patients use the nearest child and adolescent psychiatric outpatient clinic for convenience and because child and adolescent psychiatric outpatient clinics are expected to deliver equal services of equal quality (111). There is no competitive market in this part of the health sector. Furthermore, it is unlikely that patients would sort in response to provider preferences, as the

For peer review only - http://bmjopen.bmj.com/site/about/guidelines.xhtml

variation in rates of ADHD diagnosis and treatment are not common knowledge among patients or their parents. Consequently, children with similar ADHD symptoms are *as-if* randomly assigned to ADHD medication or no ADHD medication at a national level.

Next, we will control for the characteristics of the catchment area and the patient mix of each provider that may partially explain variation in diagnosis and prescription practices. We have relied on existing research and expert knowledge in our assessment of relevant control variables and included variables in our data accordingly (see e.g.112). In addition to our micro-level data, we construct a municipality-level data set from publicly available register data to further improve our adjustment for potential confounding. (Our list of potential confounders is presented in Table 1.) Exclusion is plausible due to the existence of large geographical variation in ADHD diagnosis and medication, net of demographic factors (43, 63, 113), which suggests that clinical practice varies for idiosyncratic reasons. As further validity assessment, we will analyze whether there is geographical variation in ADHD symptoms using the nationwide The Norwegian Mother and Child Cohort study (MoBa), and whether referral frequencies vary geographically using data from the Norwegian Patient Register (NPR).

Table 1. Potential confounders for facility-level instrumental variables

Confounder category	Potential confounders	Covered	Data source
Geographic	Urban/rural	Yes.	Municipality data set and municipality level covariates in microdata (Table 2).
Patient characteristics	Race, education, income, age, insurance status, health	Insurance status is not likely an issue in the Norwegian	Microdata (Table 2).

For peer review only - http://bmjopen.bmj.com/site/about/guidelines.xhtml

	status/comorbid conditions, health behaviors	health care system. Health behavior is unobserved.	
Facility characteristics	Procedure volume, facility volume, clinical services offered, departments, teaching status, profit status, trauma designation, delivery system type, practice type	Although the sizes of the facilities vary, the provided services and the organization of the facility are part of the same nationwide health care system and should be similar. Profit status is likely not an issue in Norwegian health care system.	Municipality data set and municipality level covariates in microdata (Table 2).
Treatment characteristics	Receipt of other treatments	We may adjust for contacts with health services for treatment and common prescriptions (e.g., melatonin, antidepressants)	Microdata (Table 2).

Table based on Garabedian et al., 2016 (112)

Triangulation and statistical methodology

Our assessment of causal effects relies on IV triangulated with other methods based on other assumptions (114). As pointed out by Matthay et al. (115): "All methods involve untestable assumptions and tradeoffs in precision and validity" and there is seldom a "… clear justification for exclusive reliance on one method". We will supplement our IV-analysis with mixed-effects models, propensity score matching (PSM), inverse-probability weighting and fixed-effects regression (for medication) (97). These are also the methods we will base our analysis on if IV-conditions are not met. All analyses will be performed in Stata (116) and R (117).

For peer review only - http://bmjopen.bmj.com/site/about/guidelines.xhtml

Data and study sample

Our data comprises one sample of patients and one sample of controls from the general population. The patient sample includes all persons between 5 and 18 years of age that had any contact (for any reason) with child and adolescent psychiatric outpatient clinics between 2009-2011 (i.e., birth cohorts 1991-2006). Children younger than 5 years are not included as few children are diagnosed with ADHD before age 5 (43). The control sample is a random sample from the general population of equal size as the patient sample defined as individuals in Norway 5-18 years old without reported contact (for any reason) with child and adolescent psychiatric outpatient clinics in 2009-2011. The control sample is included to enable us to compare the prognosis among patients diagnosed with ADHD and patients in contact with child and adolescent psychiatric outpatient clinics to the general population.

As the project will examine outcomes that occur at different points over a life course (injuries, contact with child welfare services, contact with adult psychiatric health services, education, income, welfare dependency, comorbidity, crime, self-harm, suicide, and mortality), we will receive data for 2009-2019 in 2020 and for 2020-2021 in 2022. We have estimated that the patient sample with an equally sized control sample from the general population (matched on age, gender and geography) will be approximately 200 000. All analyses are registry-based. We combine data from a total of nine national registries, presented in Table 2. (Abbreviations in parentheses.)

Table 2. Data from registries included in the project

National register	Variables

For peer review only - http://bmjopen.bmj.com/site/about/guidelines.xhtml

Norwegian Patient Register (NPR)	Diagnoses (ICD-10, all F-diagnoses by multiaxial coding), institution, in/out date, waiting time code, contact type, referral from institution, referral type, referral reason, injury (specialist care), injury severity, child welfare service involvement, child's care situation, child's environment, gender, age, patient catchment area
Control and Payment of Health Reimbursement Register (KUHR)	Injuries (emergency room) ICPC-2 diagnoses (somatic: fracture, concussion, eye injuries, penetration, burn, poisoning, other injuries/ psychiatric: suicide attempt, hyperkinetic disorder, anorexia, schizophrenia)
Norwegian Cause of Death Registry (NCoDR)	Death, cause of death (three categories: suicide, injury, other)
Norwegian Prescription Database (NorPD)	Psychostimulants for ADHD (ATC-codes N06B, CO2, CO2A), melatonin (ATC-codes N05, N05B), antidepressants (ATC-code N06A), antiepileptics (ATC-code N03A), defined daily dose (DDD)
The Central Penal and Police Register (Strasak)	Criminal charges (ten categories: property theft, other offences for profit, criminal damage, violence and maltreatment, sexual offences, drug and alcohol offences, public order and integrity violations, traffic offences, other offences)
The Central Population Register (CPR)	Country of origin (world region), parents' marital status, emigration
Municipality-State-Reporting (KOSTRA)	Case type, who reported case, content of case, measure in child welfare services law, removal of custody, reason for administrative decision
Norwegian Education Database (NUDB)	Highest completed level of education, parents' educational level, ended high school education before completion ("dropout"), average grade level for primary school, grades from high school completion, national test scores, order/conduct, absence
Income, tax and wealth register	Income, work assessment benefit (AAP), social assistance, disability pension benefit, unemployment assistance ("dagpenger"), parents' income

For peer review only

The registry data set will be supplemented with a municipality data set based on publicly available aggregated administrative data. The municipalities will be grouped by child and adolescent psychiatric outpatient clinic catchment areas. The municipality data set serves two purposes: (i) examine associations between environmental factors and geographical variation in ADHD diagnosis and prescription patterns, and (ii) account for population mix at child and adolescent psychiatric outpatient clinics in analyses. The municipality data set will include covariates for socioeconomic conditions (education, income, welfare recipiency), crime, income inequality, living conditions, health service utilization, and health service economic variables.

Patient and public involvement

Patients and public were not involved in the development of work packages or study design.

Discussion

This project examines (i) the prognosis of ADHD, (ii) within-country variation in ADHD diagnosis and medication rates by clinics' catchment area, (iii) causal effects of being diagnosed with ADHD on long-term outcomes and (iv) causal effects of ADHD medication on long-term outcomes. The project relies on Norwegian nationwide register data and employs several methodological approaches, including survival analysis, mixed-effects models, and instrumental variables.

As outlined in the introduction, the debate concerning the long-term consequences of variation in diagnosis and medication of ADHD is far from settled. RCTs on ADHD may not adequately address the controversy on long-term effects of ADHD diagnosis or medication, as randomization of these exposures on a large scale with long follow-up is infeasible. Causal inference from conventional epidemiological designs is also generally implausible, due to

For peer review only - http://bmjopen.bmj.com/site/about/guidelines.xhtml

unobserved confounding. By combining rich nationwide register data with an instrumental variables design, we are able to circumvent the shortcomings of both RCTs and conventional epidemiological designs on these topics. Our approach aims to fill this knowledge gap by analyzing effects of diagnosis and medication for children and adolescents on long-term outcomes.

In this sense, this project has merit to provide empirical evidence that neither RCTs nor conventional epidemiological designs can address. First, there is to our knowledge no trial randomizing children to ADHD diagnosis. Our approach aims to fill this knowledge gap by instrument variable analysis for children at the margin of an ADHD diagnosis. Second, most clinical trials of medication in ADHD to date has been conducted on children that clearly meet diagnostic criteria, whereas the variation and controversy is regarding children on the margin of receiving medication.

This project is the first detailed examination of within-country variation in ADHD diagnosis and ADHD medication by child and adolescent outpatient psychiatric clinics' catchment areas, including possible associations with socioeconomic and demographic covariates. The project will be the first application of provider preference for ADHD diagnosis as an instrumental variable. The project will also be the first application of instrumental variables to assess the effects of ADHD diagnosis and ADHD medication on several psychosocial outcomes.

The project is subject to the following limitations: Register data is limited to pre-collected data in registers, which also determine data selection and data quality (118). Register data can be considered as the "administrator's view of the world" which affects data definitions and data collection practice, and often makes it challenging to understand data-generating processes (118). Moreover, register data are generally considered reliable, but the validity is important to assess due to complex data collection processes that varies between registers (93, 119).

For peer review only - http://bmjopen.bmj.com/site/about/guidelines.xhtml

Ethics and dissemination

Data management

The Norwegian Prescription Database (NorPD) will combine data from the different registries and replace the personal identification number with a project-specific number (pseudonymization) before distributing data to the research group to protect the privacy of participants. NorPD will keep the linkage key between the personal identification number and the project-specific number. Data will be stored and analyzed on a secure server at Haukeland University Hospital that only the PI and other pre-approved researchers will have access to. To ensure the quality and reproducibility of our data management and statistical analyses, we will (i) cross-check each step in the data cleaning and preparation process, and (ii) document each step from the raw data to the final data sets and statistical analyses in version-controlled syntax and log-files. Missing data will be assessed, and we will consider strategies for multiple imputation.

Ethics approval and consent to participate: The project is approved by the Regional Committee for Medical and Health Research Ethics, Norway: REC South East, Committee D (REC number 2017/2150/REC south-east D). Due to the large number of participants, it is not feasible to obtain consent from all participants. The study has been evaluated by REC to be "of considerable importance to society" and to "protect the integrity and welfare of the participants". Based on the fulfillment of these criteria, the project has been exempted from the requirement of collecting informed consent from participants. The REC approval is given under the precondition that the project is conducted as described in the project application, protocol, communication, and the administrative provisions in the Norwegian Health Research Act ("Helseforskningsloven") and appurtenant regulations.

Availability of data and materials: The data used in this project may not be made publicly available due to the sensitive nature of the data. Current approvals and regulations do not allow

For peer review only

For peer review only - http://bmjopen.bmj.com/site/about/guidelines.xhtml

sharing these data. Due to personal data protection regulations (GDPR), restrictions in the ethics approval and restrictions by the registry owners, data may not be shared. We may share statistical code.

Dissemination: The publication strategy distinguishes between six types of publications planned from this project:

a. Preparatory publications: This includes the present protocol paper, trial registrations, systematic literature reviews on subject matter issues regarding ADHD (120) and provider preference IV applications (121).

b. WP1: The epidemiology of ADHD prognosis. We will utilize all data to describe common trajectories in ADHD. Multiple comparison groups will be employed including other patients referred to child and adolescent psychiatry who got other diagnoses or no diagnosis, and also the general population. Outcomes are according to data as described in Table 2.

c. WP2: The variation in ADHD diagnosis and treatment. We will describe in detail the geographical variation in ADHD diagnosis and medication, and also explore if characteristics of the catchment areas may explain some of the observed variation in clinical practice. We will publish results from the survey among clinicians at the public sector child and adolescent psychiatric outpatient clinics in Norway, exploring if their opinions and attitudes to ADHD diagnosis and treatment vary from restrictive to liberal, and if this variation corresponds with the observed geographical variation in rates of ADHD diagnosis and medication. Further, we will publish results from the health survey, exploring if there is an association between rates of ADHD symptoms and ADHD diagnosis and medication.

d. WP3: Long-term effects of receiving an ADHD diagnosis for children on the margin of the diagnostic threshold. Publications on the effect of ADHD diagnosis among those referred to child and adolescent psychiatry on outcomes as described above. Outcomes according to data in Table 2.

For peer review only - http://bmjopen.bmj.com/site/about/guidelines.xhtml

e. WP4: Long-term effects of ADHD medication. Publications on the effect of ADHD medication among those diagnosed with ADHD on outcomes as described above. Outcomes according to data in Table 2.

f. Exploratory publications on hypotheses and topics beyond those here described. These may be subject to additional approvals from the ethics committee and the owners of the registries, eventually also an update of the Data Protection Impact Assessment.

We will follow International Committee of Medical Journal Editors (ICMJE) Vancouver recommendations for authorship when publishing the papers. Most of our team members have significant experience in writing and publishing scientific papers, and we believe our multidisciplinary team's experience will serve as the main expertise in writing the planned articles. We aim to publish with open access. Substantial amendments to our protocol will be reported to relevant parties through relevant channels.

For peer review only

For peer review only - http://bmjopen.bmj.com/site/about/guidelines.xhtml

References

1. Polanczyk GV, Salum GA, Sugaya LS, Caye A, Rohde LA. Annual research review: A meta-analysis of the worldwide prevalence of mental disorders in children and adolescents. J Child Psychol Psychiatry. 2015;56(3):345-65.
2. Polanczyk GV, Willcutt EG, Salum GA, Kieling C, Rohde LA. ADHD prevalence estimates across three decades: an updated systematic review and meta-regression analysis. International Journal of Epidemiology. 2014;43(2):434-42.
3. Polanczyk G, de Lima MS, Horta BL, Biederman J, Rohde LA. The Worldwide Prevalence of ADHD: A Systematic Review and Metaregression Analysis. Am J Psychiat. 2007;164(6):942-8.
4. Thomas R, Sanders S, Doust J, Beller E, Glasziou P. Prevalence of attention-deficit/hyperactivity disorder: a systematic review and meta-analysis. Pediatrics. 2015;135(4):e994-1001.
5. Franke B, Michelini G, Asherson P, Banaschewski T, Bilbow A, Buitelaar JK, et al. Live fast, die young? A review on the developmental trajectories of ADHD across the lifespan. European neuropsychopharmacology : the journal of the European College of Neuropsychopharmacology. 2018;28(10):1059-88.
6. American Psychiatric Association. Diagnostic and statistical manual of mental disorders : DSM-5. 5th ed. ed. Washington, D.C: American Psychiatric Association; 2013.
7. Faraone SV, Asherson P, Banaschewski T, Biederman J, Buitelaar JK, Ramos-Quiroga JA, et al. Attention-deficit/hyperactivity disorder. Nat Rev Dis Primers. 2015;1:23.
8. Shaw M, Hodgkins P, Caci H, Young S, Kahle J, Woods AG, et al. A systematic review and analysis of long-term outcomes in attention deficit hyperactivity disorder: effects of treatment and non-treatment. BMC Med. 2012;10:99.
9. Dalsgaard S, Leckman JF, Mortensen PB, Nielsen HS, Simonsen M. Effect of drugs on the risk of injuries in children with attention deficit hyperactivity disorder: a prospective cohort study. The Lancet Psychiatry. 2015;2(8):702-9.
10. Dalsgaard S, Mortensen PB, Frydenberg M, Maibing CM, Nordentoft M, Thomsen PH. Association between Attention-Deficit Hyperactivity Disorder in childhood and schizophrenia later in adulthood. Eur Psychiat. 2014;29(4):259-63.
11. Barbaresi WJ, Colligan RC, Weaver AL, Voigt RG, Killian JM, Katusic SK. Mortality, ADHD, and Psychosocial Adversity in Adults With Childhood ADHD: A Prospective Study. Pediatrics. 2013;131(4).
12. Allely CS. The association of ADHD symptoms to self-harm behaviours: a systematic PRISMA review. BMC Psychiatry. 2014;14(1):133.
13. Chen VC-H, Chan H-L, Wu S-I, Lee M, Lu M-L, Liang H-Y, et al. Attention-Deficit/Hyperactivity Disorder and Mortality Risk in Taiwan. JAMA Network Open. 2019;2(8):e198714-e.
14. London AS, Landes SD. Attention Deficit Hyperactivity Disorder and adult mortality. Preventive medicine. 2016;90:8-10.
15. Klein B, Damiani-Taraba G, Koster A, Campbell J, Scholz C. Diagnosing attention-deficit hyperactivity disorder (ADHD) in children involved with child protection services: are current diagnostic guidelines acceptable for vulnerable populations? Child Care Health Dev. 2015;41(2):178-85.
16. Zito JM, Safer DJ, Sai D, Gardner JF, Thomas D, Coombes P, et al. Psychotropic Medication Patterns Among Youth in Foster Care. Pediatrics. 2008;121(1):e157-e63.
17. Klein RG, Mannuzza S, Olazagasti MAR, Roizen E, Hutchison JA, Lashua EC, et al. Clinical and Functional Outcome of Childhood Attention-Deficit/Hyperactivity Disorder 33 Years Later. Archives of General Psychiatry. 2012;69(12):1295-303.
18. Fletcher J. The effects of childhood ADHD on adult labor market outcomes. Cambridge, MA: National Bureau of Economic Research; 2013. Contract No.: 18689.
19. Chang Z, Lichtenstein P, Halldner L, D'Onofrio B, Serlachius E, Fazel S, et al. Stimulant ADHD medication and risk for substance abuse. Journal of Child Psychology and Psychiatry. 2014;55(8):878-85.

For peer review only - http://bmjopen.bmj.com/site/about/guidelines.xhtml

20. Dalsgaard S, Mortensen PB, Frydenberg M, Thomsen PH. ADHD, stimulant treatment in childhood and subsequent substance abuse in adulthood - A naturalistic long-term follow-up study. Addictive Behaviors. 2014;39(1):325-8.
21. Madsen AG, Dalsgaard S. Prevalence of smoking, alcohol and substance use among adolescents with attention-deficit/hyperactivity disorder in Denmark compared with the general population. Nordic Journal of Psychiatry. 2014;68(1):53-9.
22. Erskine HE, Norman RE, Ferrari AJ, Chan GC, Copeland WE, Whiteford HA, et al. Long-Term Outcomes of Attention-Deficit/Hyperactivity Disorder and Conduct Disorder: A Systematic Review and Meta-Analysis. J Am Acad Child Adolesc Psychiatry. 2016;55(10):841-50.
23. Fletcher J, Wolfe B. Long-term consequences of childhood ADHD on criminal activities. J Ment Health Policy Econ. 2009;12(3):119-38.
24. Dalsgaard S. Long-term psychiatric and criminality outcome of children with attention-deficit/hyperactivity disorder. Nordic Journal of Psychiatry. 2003;57(2):160-.
25. National Institute for Health and Care Excellence. Attention deficit hyperactivity disorder: diagnosis and management. NICE guideline [N87]2018.
26. Helsedirektoratet. ADHD/Hyperkinetisk forstyrrelse - Nasjonal faglig retningslinje for utredning, behandling og oppfølging. 2017.
27. Daley D, Van Der Oord S, Ferrin M, Cortese S, Danckaerts M, Doepfner M, et al. Practitioner review: current best practice in the use of parent training and other behavioural interventions in the treatment of children and adolescents with attention deficit hyperactivity disorder. Journal of Child Psychology and Psychiatry. 2018;59(9):932-47.
28. Posner J, Polanczyk GV, Sonuga-Barke E. Attention-deficit hyperactivity disorder. Lancet (London, England). 2020;395(10222):450-62.
29. Cortese S, Adamo N, Del Giovane C, Mohr-Jensen C, Hayes AJ, Carucci S, et al. Comparative efficacy and tolerability of medications for attention-deficit hyperactivity disorder in children, adolescents, and adults: a systematic review and network meta-analysis. The Lancet Psychiatry. 2018;5(9):727-38.
30. Biederman J, Mick E, Faraone SV. Age-dependent decline of symptoms of attention deficit hyperactivity disorder: impact of remission definition and symptom type. Am J Psychiatry. 2000;157(5):816-8.
31. Hinshaw SP, Arnold LE. Attention-deficit hyperactivity disorder, multimodal treatment, and longitudinal outcome: evidence, paradox, and challenge. Wiley interdisciplinary reviews Cognitive science. 2015;6(1):39-52.
32. Storebo OJ, Krogh HB, Ramstad E, Moreira-Maia CR, Holmskov M, Skoog M, et al. Methylphenidate for attention-deficit/hyperactivity disorder in children and adolescents: Cochrane systematic review with meta-analyses and trial sequential analyses of randomised clinical trials. BMJ (Clinical research ed). 2015;351:h5203.
33. Cortese S. Debate: Are Stimulant Medications for Attention-Deficit/Hyperactivity Disorder Effective in the Long Term? Journal of the American Academy of Child and Adolescent Psychiatry. 2019;58(10):936.
34. Coghill D. Debate: Are Stimulant Medications for Attention-Deficit/Hyperactivity Disorder Effective in the Long Term?(For). 2019.
35. Swanson JM. Debate: Are Stimulant Medications for Attention-Deficit/Hyperactivity Disorder Effective in the Long Term?(Against). 2019.
36. Pliszka SR. Is There Long-Term Benefit From Stimulant Treatment for ADHD? Am J Psychiat. 2019;176(9):685-6.
37. Chang Z, Ghirardi L, Quinn PD, Asherson P, D'Onofrio BM, Larsson H. Risks and benefits of ADHD medication on behavioral and neuropsychiatric outcomes: A qualitative review of pharmacoepidemiology studies using linked prescription databases. Biological psychiatry. 2019;86(5):335.

For peer review only - http://bmjopen.bmj.com/site/about/guidelines.xhtml

38. Lichtenstein P, Halldner L, Zetterqvist J, Sjolander A, Serlachius E, Fazel S, et al. Medication for attention deficit-hyperactivity disorder and criminality. The New England journal of medicine. 2012;367(21):2006-14.
39. Dalsgaard S, Mortensen PB, Frydenberg M, Thomsen PH. Long-term criminal outcome of children with attention deficit hyperactivity disorder. Criminal behaviour and mental health : CBMH. 2013;23(2):86-98.
40. Lundstrom S, Forsman M, Larsson H, Kerekes N, Serlachius E, Langstrom N, et al. Childhood neurodevelopmental disorders and violent criminality: a sibling control study. J Autism Dev Disord. 2014;44(11):2707-16.
41. Mohr-Jensen C, Müller Bisgaard C, Boldsen SK, Steinhausen H-C. Attention-Deficit/Hyperactivity Disorder in Childhood and Adolescence and the Risk of Crime in Young Adulthood in a Danish Nationwide Study. Journal of the American Academy of Child & Adolescent Psychiatry. 2019;58(4):443-52.
42. Quinn PD, Chang Z, Hur K, Gibbons RD, Lahey BB, Rickert ME, et al. ADHD Medication and Substance-Related Problems. Am J Psychiat.0(0):appi.ajp.2017.16060686.
43. Dalsgaard S, Nielsen HS, Simonsen M. Consequences of ADHD medication use for children's outcomes. Journal of Health Economics. 2014;37:137-51.
44. Chang Z, Lichtenstein P, Deonofrio BM, Sjolander A, Larsson H. Serious transport accidents in adults with attention-deficit/hyperactivity disorder and the effect of medication: a population-based study.(Report). JAMA Psychiatry. 2014;71(3):319.
45. van de Loo-Neus GH, Rommelse N, Buitelaar JK. To stop or not to stop? How long should medication treatment of attention-deficit hyperactivity disorder be extended? European neuropsychopharmacology : the journal of the European College of Neuropsychopharmacology. 2011;21(8):584-99.
46. Getahun D, Jacobsen SJ, Fassett MJ, Chen W, Demissie K, Rhoads GG. Recent trends in childhood attention-deficit/hyperactivity disorder. JAMA Pediatrics. 2013;167(3):282-8.
47. Atladottir HO, Gyllenberg D, Langridge A, Sandin S, Hansen SN, Leonard H, et al. The increasing prevalence of reported diagnoses of childhood psychiatric disorders: a descriptive multinational comparison. Eur Child Adolesc Psychiatry. 2015;24(2):173-83.
48. Stephenson CP, Karanges E, McGregor IS. Trends in the utilisation of psychotropic medications in Australia from 2000 to 2011. The Australian and New Zealand journal of psychiatry. 2013;47(1):74-87.
49. Man KKC, Ip P, Hsia Y, Chan EW, Chui CSL, Lam MPS, et al. ADHD Drug Prescribing Trend Is Increasing Among Children and Adolescents in Hong Kong. Journal of attention disorders. 2017;21(14):1161-8.
50. Polanczyk GV, Salum GA, Sugaya LS, Caye A, Rohde LA. Annual Research Review: A meta-analysis of the worldwide prevalence of mental disorders in children and adolescents. Journal of Child Psychology and Psychiatry. 2015;56(3):345-65.
51. Thomas R, Sanders S, Doust J, Beller E, Glasziou P. Prevalence of Attention-Deficit/Hyperactivity Disorder: A Systematic Review and Meta-analysis. Pediatrics2015. p. E994-E1001.
52. Sayal K, Prasad V, Daley D, Ford T, Coghill D. ADHD in children and young people: prevalence, care pathways, and service provision. The Lancet Psychiatry. 2017.
53. Faraone SV, Sergeant JA, Gillberg C, Biederman J, Clinical N. The Worldwide prevalence of ADHD: is it an American condition? World Psychiatry. 2003;2(2):104-13.
54. Raman SR, Man KKC, Bahmanyar S, Berard A, Bilder S, Boukhris T, et al. Trends in attention-deficit hyperactivity disorder medication use: a retrospective observational study using population-based databases. The Lancet Psychiatry.
55. Zoega H, Furu K, Halldorsson M, Thomsen PH, Sourander A, Martikainen JE. Use of ADHD drugs in the Nordic countries: a population-based comparison study. Acta Psychiatrica Scandinavica. 2011;123(5):360-7.

For peer review only - http://bmjopen.bmj.com/site/about/guidelines.xhtml

56. Piper BJ, Ogden CL, Simoyan OM, Chung DY, Caggiano JF, Nichols SD, et al. Trends in use of prescription stimulants in the United States and Territories, 2006 to 2016. PloS one. 2018;13(11):e0206100-e.
57. McDonald DC, Jalbert SK. Geographic variation and disparity in stimulant treatment of adults and children in the United States in 2008. Psychiatric services (Washington, DC). 2013;64(11):1079-86.
58. Fulton BD, Scheffler RM, Hinshaw SP, Levine P, Stone S, Brown TT, et al. National variation of ADHD diagnostic prevalence and medication use: health care providers and education policies. Psychiatr Serv. 2009;60(8):1075-83.
59. Madsen KB, Ersbøll AK, Olsen J, Parner E, Obel C. Geographic analysis of the variation in the incidence of ADHD in a country with free access to healthcare: a Danish cohort study. International journal of health geographics. 2015;14:24-.
60. Wallach-Kildemoes H, Skovgaard AM, Thielen K, Pottegard A, Mortensen LH. Social adversity and regional differences in prescribing of ADHD medication for school-age children. Journal of developmental and behavioral pediatrics : JDBP. 2015;36(5):330-41.
61. Hjern A, Weitoft GR, Lindblad F. Social adversity predicts ADHD-medication in school children--a national cohort study. Acta paediatrica (Oslo, Norway : 1992). 2010;99(6):920-4.
62. Knopf H, Holling H, Huss M, Schlack R. Prevalence, determinants and spectrum of attention-deficit hyperactivity disorder (ADHD) medication of children and adolescents in Germany: results of the German Health Interview and Examination Survey (KiGGS). BMJ Open. 2012;2(6).
63. Ørstavik R, Gustavson K, Rohrer-Baumgartner N, Biele G, Furu K, Karlstad Ø, et al. ADHD i Norge: En statusrapport. www.fhi.no: Folkehelseinstituttet; 2016.
64. Surén P, Bakken IJ, Lie KK, Schjølberg S, Aase H, Reichborn-Kjennerud T, et al. Differences across counties in the registered prevalence of autism, ADHD, epilepsy and cerebral palsy in Norway. Tidsskr Nor Laegeforen. 2013;133(18):1929-34.
65. Paul Kjetel Soldal Lillemoen SRK, Steinar Hunskår, Sabine Ruths. Prescriptions for ADHD medication, 2004 – 08. Tidsskriftet. 2012.
66. Evans WN, Morrill MS, Parente ST. Measuring inappropriate medical diagnosis and treatment in survey data: The case of ADHD among school-age children. Journal of Health Economics. 2010;29(5):657-73.
67. Karlstad O, Furu K, Stoltenberg C, Haberg SE, Bakken IJ. ADHD treatment and diagnosis in relation to children's birth month: Nationwide cohort study from Norway. Scand J Public Health. 2017;45(4):343-9.
68. Surén P, Thorstensen AG, Tørstad M, Emhjellen PE, Furu K, Biele G, et al. Diagnosis of hyperkinetic disorder among children in Norway. Tidsskr Nor Legeforen. 2018.
69. Frances A. The past, present and future of psychiatric diagnosis. World Psychiatry. 2013;12(2).
70. Lebowitz MS. Stigmatization of ADHD: A Developmental Review. J Atten Disord. 2016;20(3):199-205.
71. Lakhan SE, Kirchgessner A. Prescription stimulants in individuals with and without attention deficit hyperactivity disorder: misuse, cognitive impact, and adverse effects. Brain and behavior. 2012;2(5):661-77.
72. Kooij JJS, Bijlenga D, Salerno L, Jaeschke R, Bitter I, Balázs J, et al. Updated European Consensus Statement on diagnosis and treatment of adult ADHD. Eur Psychiat. 2019;56:14-34.
73. Elsabbagh M, Divan G, Koh YJ, Kim YS, Kauchali S, Marcin C, et al. Global prevalence of autism and other pervasive developmental disorders. Autism research : official journal of the International Society for Autism Research. 2012;5(3):160-79.
74. Collishaw S. Annual Research Review: Secular trends in child and adolescent mental health. Journal of Child Psychology and Psychiatry. 2015;56(3):370-93.
75. Frances A. Saving normal : an insider's revolt against out-of-control psychiatric diagnosis, DSM-5, Big Pharma, and the medicalization of ordinary life. New York: William Morrow; 2013.
76. Atkinson M, Hollis CJAoDiC-E, Practice. NICE guideline: attention deficit hyperactivity disorder. 2010;95(1):24-7.

For peer review only - http://bmjopen.bmj.com/site/about/guidelines.xhtml

77. Speerforck S, Stolzenburg S, Hertel J, Grabe HJ, Strauß M, Carta MG, et al. ADHD, stigma and continuum beliefs: A population survey on public attitudes towards children and adults with attention deficit hyperactivity disorder. Psychiatry Res. 2019;282:112570.
78. McLennan JD. Understanding attention deficit hyperactivity disorder as a continuum. Can Fam Physician. 2016;62(12):979-82.
79. Larsson H, Anckarsater H, Rastam M, Chang Z, Lichtenstein P. Childhood attention-deficit hyperactivity disorder as an extreme of a continuous trait: a quantitative genetic study of 8,500 twin pairs. Journal of child psychology and psychiatry, and allied disciplines. 2012;53(1):73-80.
80. Owens J. Relationships between an ADHD Diagnosis and Future School Behaviors among Children with Mild Behavioral Problems. 2020;0(0):0038040720909296.
81. Eccleston L, Williams J, Knowles S, Soulsby L. Adolescent experiences of living with a diagnosis of ADHD: a systematic review and thematic synthesis. Emotional and Behavioural Difficulties. 2019;24(2):119-35.
82. Ringer N. Living with ADHD: A Meta-Synthesis Review of Qualitative Research on Children's Experiences and Understanding of Their ADHD. International Journal of Disability, Development and Education. 2020;67(2):208-24.
83. Lebowitz MS. Stigmatization of ADHD: A Developmental Review. Journal of attention disorders. 2016;20(3):199-205.
84. Mueller AK, Fuermaier ABM, Koerts J, Tucha L. Stigma in attention deficit hyperactivity disorder. Atten Defic Hyperact Disord. 2012;4(3):101-14.
85. Brookhart MA, Schneeweiss S. Preference-based instrumental variable methods for the estimation of treatment effects: assessing validity and interpreting results. Int J Biostat. 2007;3(1):Article 14.
86. Fang G, Brooks JM, Chrischilles EA. A new method to isolate local-area practice styles in prescription use as the basis for instrumental variables in comparative effectiveness research. Med Care. 2010;48(8):710-7.
87. Ministry of Health and Care Services. Meld. St. 7 (2019-2020) National Health and Hospital Plan 2020-2023. 2019.
88. Magnus P, Birke C, Vejrup K, Haugan A, Alsaker E, Daltveit AK, et al. Cohort profile update: the Norwegian mother and child cohort study (MoBa). International journal of epidemiology. 2016;45(2):382-8.
89. Chorniy A, Kitashima L. Sex, drugs, and ADHD: The effects of ADHD pharmacological treatment on teens' risky behaviors. Labour Economics. 2016;43(Supplement C):87-105.
90. Hernán MA, Hsu J, Healy B. A Second Chance to Get Causal Inference Right: A Classification of Data Science Tasks. CHANCE. 2019;32(1):42-9.
91. Moser A, Puhan MA, Zwahlen M. The role of causal inference in health services research I: tasks in health services research. Int J Public Health. 2020;65(2):227-30.
92. De Montgomery CJ, Smith Jervelund S. Nordic registry data – part II: health, employment and children. 2020;48(2):123-4.
93. Smith Jervelund S, De Montgomery CJ. Nordic registry data: value, validity and future. 2020;48(1):1-4.
94. Kirkwood BR. Essential Medical Statistics. 2nd ed.. ed: Hoboken: John Wiley & Sons, Incorporated; 2003.
95. Schuster NA, Hoogendijk EO, Kok AAL, Twisk JWR, Heymans MW. Ignoring competing events in the analysis of survival data may lead to biased results: a non-mathematical illustration of competing risk analysis. Journal of Clinical Epidemiology. 2020.
96. Herle M, Micali N, Abdulkadir M, Loos R, Bryant-Waugh R, Hübel C, et al. Identifying typical trajectories in longitudinal data: modelling strategies and interpretations. Eur J Epidemiol. 2020.
97. Gelman A, Hill J. Data analysis using regression and multilevel/hierarchical models. Cambridge: Cambridge University Press; 2007.
98. Stukel TA, Fisher ES, Wennberg DE, Alter DA, Gottlieb DJ, Vermeulen MJ. Analysis of observational studies in the presence of treatment selection bias: effects of invasive cardiac

For peer review only - http://bmjopen.bmj.com/site/about/guidelines.xhtml

management on AMI survival using propensity score and instrumental variable methods. Jama. 2007;297(3):278-85.
99. Murnane RJ, Willett JB. Methods matter: Improving causal inference in educational research. Oxford, New York: Oxford University Press; 2010.
100. Stukel TA, Fisher ES, Wennberg DE, Alter DA, Gottlieb DJ, Vermeulen MJ. Analysis of observational studies in the presence of treatment selection bias: effects of invasive cardiac management on AMI survival using propensity score and instrumental variable methods. Jama. 2007;297(3):278-85.
101. McClellan M, McNeil BJ, Newhouse JP. Does more intensive treatment of acute myocardial infarction in the elderly reduce mortality? Analysis using instrumental variables. Jama. 1994;272(11):859-66.
102. Cook TD, Shadish WR, Wong VC. Three conditions under which experiments and observational studies produce comparable causal estimates: New findings from within-study comparisons. 2008;27(4):724-50.
103. Potter BJ, Dormuth C, Le Lorier J. A theoretical exploration of therapeutic monomania as a physician-based instrumental variable. Pharmacoepidemiology and drug safety. 2019.
104. Rassen JA, Brookhart MA, Glynn RJ, Mittleman MA, Schneeweiss S. Instrumental variables II: instrumental variable application—in 25 variations, the physician prescribing preference generally was strong and reduced covariate imbalance. Journal of Clinical Epidemiology. 2009;62(12):1233-41.
105. Ionescu-Ittu R, Abrahamowicz M, Pilote L. Treatment effect estimates varied depending on the definition of the provider prescribing preference-based instrumental variables. Journal of Clinical Epidemiology. 2012;65(2):155-62.
106. Brooks JM, Tang Y, Chapman CG, Cook EA, Chrischilles EA. What is the effect of area size when using local area practice style as an instrument? Journal of Clinical Epidemiology. 2013;66(8, Supplement):S69-S83.
107. Kitashima L, Chorniy A. ADHD Medication Effects on Primary and Secondary Student's Academic Achievement. ADHD Medication on Child Welfare. PhD. Clemson University, South Carolina, USA: Clemson University; 2017.
108. Fallesen P, Wildeman C. The Effect of Medical Treatment of Attention Deficit Hyperactivity Disorder (ADHD) on Foster Care Caseloads:Evidence from Danish Registry Data. 2015;56(3):398-414.
109. Franklin JM, Schneeweiss S, Huybrechts KF, Glynn RJ. Evaluating possible confounding by prescriber in comparative effectiveness research. Epidemiology. 2015;26(2):238-41.
110. Dunning T. Natural experiments in the social sciences : a design-based approach. Cambridge: Cambridge University Press; 2012.
111. Magnussen J, Kalseth J. Geographical distribution of resources to health services. In: Haug K, Kaarbøe OM, Olsen TE, editors. A health service without boundaries?2009.
112. Garabedian LF, Chu P, Toh S, Zaslavsky AM, Soumerai SB. Potential bias of instrumental variable analyses for observational comparative effectiveness research. Annals of internal medicine. 2014;161(2):131-8.
113. Chorniy A, Kitashima L. Sex, drugs, and ADHD: The effects of ADHD pharmacological treatment on teens' risky behaviors. Labour Economics. 2016;43:87-105.
114. Hernán MA, Robins JM. Causal Inference: What if? Boca Raton: Chapman & Hall/CRC; 2019.
115. Matthay EC, Hagan E, Gottlieb LM, Tan ML, Vlahov D, Adler NE, et al. Alternative causal inference methods in population health research: Evaluating tradeoffs and triangulating evidence. SSM - Population Health. 2020;10:100526.
116. StataCorp. Stata Statistical Software: Release 16.1. College Station, TX: StataCorp LLC; 2020.
117. R Core Team. R: A language and environment for statistical computing. Vienna, Austria: R Foundation for Statistical Computing; 2020.
118. Thygesen L, Ersbøll A. When the entire population is the sample: strengths and limitations in register-based epidemiology. Eur J Epidemiol. 2014;29(8):551-8.

For peer review only - http://bmjopen.bmj.com/site/about/guidelines.xhtml

119. Bakken IJ, Ariansen AMS, Knudsen GP, Johansen KI, Vollset SE. The Norwegian Patient Registry and the Norwegian Registry for Primary Health Care: Research potential of two nationwide health-care registries. Scand J Public Health. 2019;48(1):49-55.
120. Chaulagain A, Lyhmann I, Widding-Havneraas T, Bjelland I, Halmøy A, Elwert F, et al. A systematic meta-review of systematic reviews on Attention Deficit Hyperactivity Disorder. 2020 [Available from: https://www.crd.york.ac.uk/prospero/display_record.php?ID=CRD42020165638.
121. Widding-Havneraas T, Chaulagain A, Lyhmann I, Daae Zachrisson H, Elwert F, McDaid D, et al. Applications of preference-based instrumental variables in health research: a systematic review. 2020 [Available from: https://www.crd.york.ac.uk/prospero/display_record.php?ID=CRD42020165014.

For peer review only

For peer review only - http://bmjopen.bmj.com/site/about/guidelines.xhtml

Authors' contributions: AM proposed the idea and developed it in collaboration with TW and KR. AM is PI for the project and responsible for ethics, funding, main supervision, and data management. TW, IL, and AC are PhD candidates, TW with a dual role also as statistician. FE, PB, SM and HDZ are involved for their expertise on statistics, epidemiology, and IV-methods, and have all advised on this project. FE developed the IV-strategy in collaboration with co-authors. IB and AH advised on the clinical substance matter and on the organization of ADHD treatment delivery in Norway. KR is head of the unit where the study belongs. AM planned the structure of this protocol paper, and the first draft was written in close collaboration between TW, AC, and IL. All other collaborators have contributed to the intellectual content of the paper, edited, and approved the final manuscript.

Funding: The project is funded by the Research Council of Norway (RCN) under the FRIMEDBIO (project number 288585) for four years starting 01.11.2019 and a grant from the Western Norway Regional Health Authority (project number 912197) from 01.01.2018 to 31.12.2023. (The project period and the funding will be extended due to delays related to the Covid-19 situation and other reasons for delay.) These are governmental funding bodies and the project has undergone peer-review as part of the funding application processes. The funders had no role in study design, data collection and analysis, decision to publish, or preparation of the manuscript. The project is owned by Centre for Research and Education in Forensic Psychiatry, Haukeland University Hospital, Bergen, Norway, where the PI and PhD candidates on the project are currently employed.

Competing interests: None.

Acknowledgements: Several academic colleagues have advised on the development of the empirical and theoretical approach for this project in the context of the broader project “Current Controversies in Psychiatry”. We acknowledge (in alphabetical order) Stål Bjørkly, Centre for Research and Education in Forensic Psychiatry, Oslo University Hospital, Oslo, Norway; Beate

For peer review only - http://bmjopen.bmj.com/site/about/guidelines.xhtml

Brinchmann, Nordland Hospital Trust, Centre for Work and Mental Health, Bodø, Norway; Ian Colman, University of Ottawa, Canada; Sarah Dorrington, Institute of Psychiatry, Psychology and Neuroscience, King's College London, England; Kevin Douglas, Department of Psychology, Simon Fraser University, Canada; Simon Gilbody, University of York, York, England; Rolf Gjestad, Center for Research and Education in Forensic Psychiatry, Haukeland University Hospital, Bergen, Norway; Nicholas Glozier, Brain and Mind Research Institute, University of Sydney, Sydney, Australia; Stephen Hart, Department of Psychology, Simon Fraser University, Canada; Samuel Harvey, School of Psychiatry, University of New South Wales, Sydney, Australia; Hallvard Lund-Heimark, NORMENT Centre of Excellence, Haukeland University Hospital, Bergen, Norway; Matthew Hotopf, Institute of Psychiatry, Psychology and Neuroscience, King's College London, England; Erik Johnsen, Division of Mental Health and NORMENT Centre of Excellence, Haukeland University Hospital, Bergen, Norway; Eoin Killackey, Orygen & Centre for Youth Mental Health, University of Melbourne, Australia; Caroline Logan, National Health Service, England; Liv Mellesdal, Division of Mental Health, Haukeland University Hospital, Bergen, Norway; Tom Palmstierna, Department of Clinical Neuroscience, Karolinska Institute, Stockholm, Sweden; Mette Senneseth, Center for Research and Education in Forensic Psychiatry, Haukeland University Hospital, Bergen, Norway; Rob Stewart, Institute of Psychiatry, Psychology and Neuroscience, King's College London, England; for valuable advice and contributions in the process of developing this project in the broader framework "Current controversies in psychiatry".

For peer review only

For peer review only - http://bmjopen.bmj.com/site/about/guidelines.xhtml

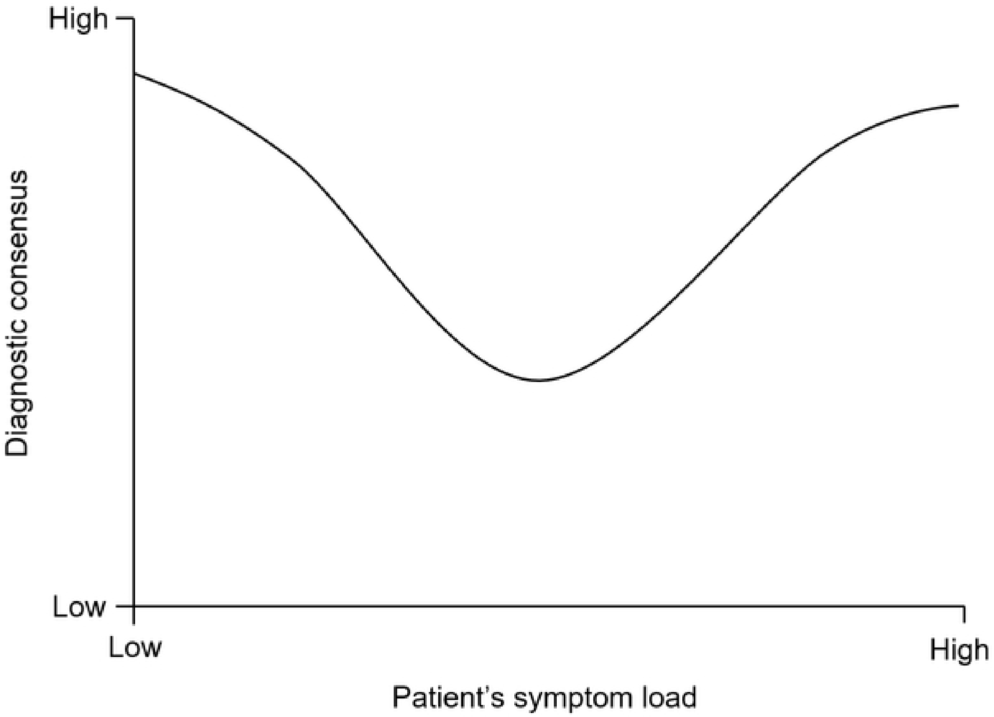

Theoretical model on clinicians' consensus on diagnosis and medication by ADHD symptom load among referred patients.

51x36mm (300 x 300 DPI)

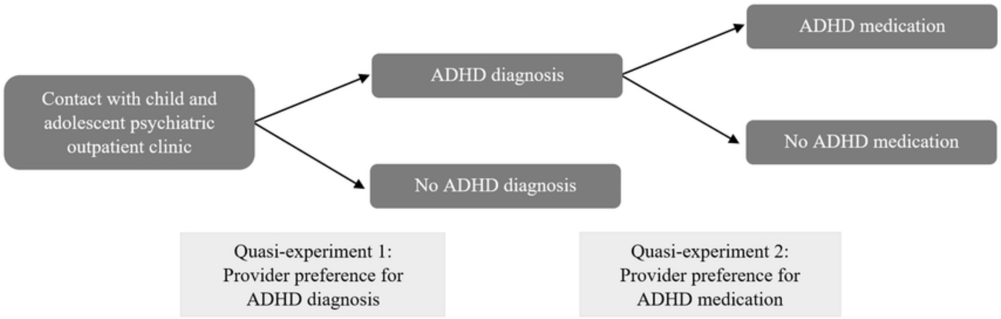

Two sources of as-if randomization. Quasi-experiment 1 is provider preference for ADHD diagnosis. Quasi-experiment 2 is provider preference for ADHD medication among those diagnosed with ADHD

72x23mm (300 x 300 DPI)

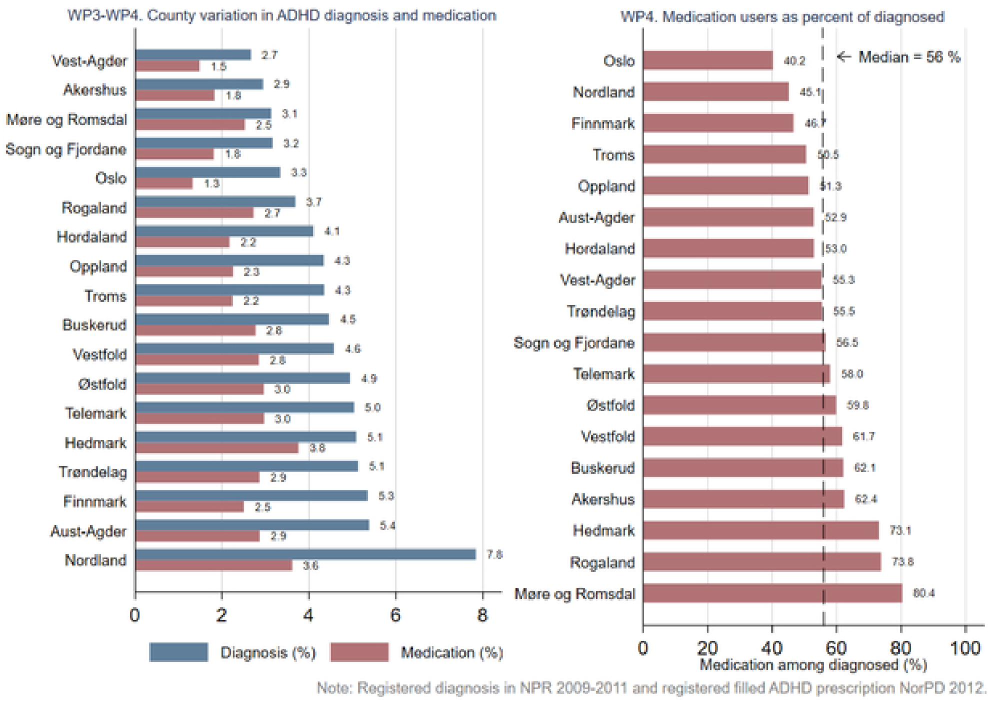

County variation in ADHD diagnosis and medication percent for children and adolescents between 10 to 19 years in Norway. ADHD as main diagnosis registered once or more times during 2009-2011.

52x38mm (300 x 300 DPI)

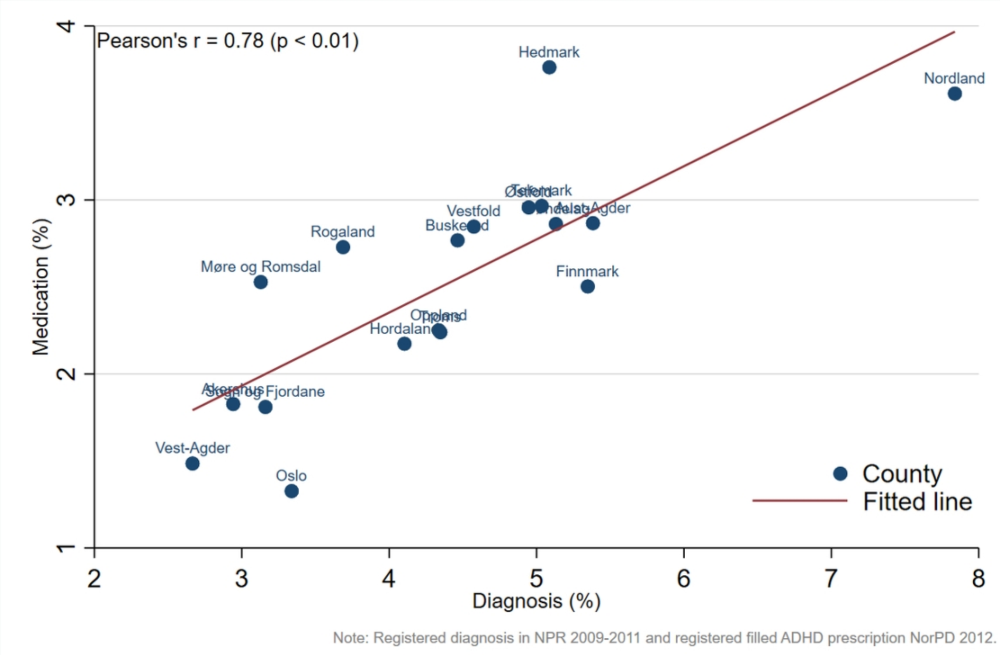

Scatterplot of ADHD diagnosis and medication among children and adolescents between 10 to 19 years by Norwegian counties. ADHD as main diagnosis registered once or more times during 2009-2011. Linear fitted line.

53x34mm (600 x 600 DPI)

STANDARD PROTOCOL ITEMS: RECOMMENDATIONS FOR INTERVENTIONAL TRIALS

SPIRIT 2013 Checklist: Recommended items to address in a clinical trial protocol and related documents*

Section/item	Item No	Description
**Administrative information**		
Title	1	Page 1
Trial registration	2a	Submitted to ISRCTN trial registry (pg. 4)
Protocol version	3	N/A
Funding	4	Funding (pg. 29)
Roles and responsibilities	5a	Page 1, Author's contribution (pg 28).
	5b	Funding, (pg. 29). Contact information: Arnstein Mykletun, Principle Investigator, email: arnstein.mykletun@uit.no (pg.2)
	5c	Funding (pg. 29).
	5d	N/A
**Introduction**		
Background and rationale	6a	Introduction (pg. 5-9).
	6b	Data and study sample (pg. 15-16) and WP3 and WP4: Long-term effects of receiving ADHD diagnosis and ADHD medication (pg. 11-12).
Objectives	7	Introduction, paragraph 12-16 (pg.9-10).
Trial design	8	N/A
**Methods: Participants, interventions, and outcomes**		
Study setting	9	Data and study sample, paragraph 1 (pg. 15-16).

For peer review only

Eligibility criteria	10	Data and study sample, paragraph 1 (pg. 15-16).
Interventions	11a	N/A
	11b	N/A
	11c	N/A
	11d	N/A
Outcomes	12	Data and study sample, paragraph 2 (pg. 16).
Participant timeline	13	Data and study sample, paragraph 1,2 (pg. 15-16).
Sample size	14	Data and study sample, paragraph 2 (pg. 16).
Recruitment	15	Data and study sample, paragraph 2 (pg. 16), Table 1 (pg. 16-17).

Methods: Assignment of interventions (for controlled trials) (N/A)

Allocation:		
Sequence generation	16a	N/A.
Allocation concealment mechanism	16b	N/A.
Implementation	16c	N/A
Blinding (masking)	17a	N/A
	17b	N/A

Methods: Data collection, management, and analysis

Data collection methods	18a	Data and study sample, paragraph 2-3 (pg.16), Table 1 (pg. 16-17).
	18b	N/A
Data management	19	Data management (pg.20).
Statistical methods	20a	Methods, paragraph 2-8 (pg. 11-15).
	20b	N/A
	20c	Data management (pg. 20).

Methods: Monitoring

Data monitoring	21a	N/A. DMC not needed as we are using register data that are already collected.
	21b	N/A
Harms	22	N/A
Auditing	23	N/A
**Ethics and dissemination**		
Research ethics approval	24	Ethics approval and consent to participate (pg. 20).
Protocol amendments	25	Dissemination, paragraph 7 (pg. 22).
Consent or assent	26a	N/A as the study is based on registry data. See Ethical approval and consent to participate (pg. 20).
	26b	N/A
Confidentiality	27	Data management (pg. 20), Ethical approval and consent to participate (pg. 20), Availability of data and materials (pg. 20-21)
Declaration of interests	28	Pg. 20,28-29.
Access to data	29	Availability of data and materials (pg. 21)
Ancillary and post-trial care	30	N/A
Dissemination policy	31a	Dissemination, paragraph 1-7 (pg. 21-22).
	31b	Dissemination, paragraph 7 (pg. 22).
	31c	Dissemination, paragraph 7 (pg.22), Availability of data and materials (pg.20-21)
**Appendices**		
Informed consent materials	32	N/A as we are using registry-based data.
Biological specimens	33	N/A

*It is strongly recommended that this checklist be read in conjunction with the SPIRIT 2013 Explanation & Elaboration for important clarification on the items. Amendments to the protocol should be tracked and dated. The SPIRIT checklist is copyrighted by the SPIRIT Group under the Creative Commons "Attribution-NonCommercial-NoDerivs 3.0 Unported" license.

For peer review only
